# Expression of *Neurog1* Instead of *Atoh1* Can Partially Rescue Organ of Corti Cell Survival

**DOI:** 10.1371/journal.pone.0030853

**Published:** 2012-01-24

**Authors:** Israt Jahan, Ning Pan, Jennifer Kersigo, Lilian E. Calisto, Ken A. Morris, Benjamin Kopecky, Jeremy S. Duncan, Kirk W. Beisel, Bernd Fritzsch

**Affiliations:** 1 University of Iowa, Department of Biology, Iowa City, Iowa, United States of America; 2 Creighton University, Department of Biomedical Sciences, Omaha, Nebraska, United States of America; Indiana University School of Medicine, United States of America

## Abstract

In the mammalian inner ear neurosensory cell fate depends on three closely related transcription factors, *Atoh1* for hair cells and *Neurog1* and *Neurod1* for neurons. We have previously shown that neuronal cell fate can be altered towards hair cell fate by eliminating *Neurod1* mediated repression of *Atoh1* expression in neurons. To test whether a similar plasticity is present in hair cell fate commitment, we have generated a knockin (KI) mouse line (*Atoh1^KINeurog1^*) in which *Atoh1* is replaced by *Neurog1*. Expression of *Neurog1* under *Atoh1* promoter control alters the cellular gene expression pattern, differentiation and survival of hair cell precursors in both heterozygous (*Atoh1^+/KINeurog1^*) and homozygous (*Atoh1^KINeurog1/KINeurog1^*) KI mice. Homozygous KI mice develop patches of organ of Corti precursor cells that express *Neurog1*, *Neurod1*, several prosensory genes and neurotrophins. In addition, these patches of cells receive afferent and efferent processes. Some cells among these patches form multiple microvilli but no stereocilia. Importantly, *Neurog1* expressing mutants differ from *Atoh1* null mutants, as they have intermittent formation of organ of Corti-like patches, opposed to a complete ‘flat epithelium’ in the absence of *Atoh1*. In heterozygous KI mice co-expression of *Atoh1* and *Neurog1* results in change in fate and patterning of some hair cells and supporting cells in addition to the abnormal hair cell polarity in the later stages of development. This differs from haploinsufficiency of *Atoh1* (*Pax2cre; Atoh1^f/+^*), indicating the effect of *Neurog1* expression in developing hair cells. Our data suggest that *Atoh1^KINeurog1^* can provide some degree of functional support for survival of organ of Corti cells. In contrast to the previously demonstrated fate plasticity of neurons to differentiate as hair cells, hair cell precursors can be maintained for a limited time by *Neurog1* but do not transdifferentiate as neurons.

## Introduction

Basic Helix-Loop-Helix (bHLH) transcription factors are essential for cell fate determination and differentiation in a wide range of tissue [1]. In the retina, spinal cord and forebrain, a mixture of bHLH expression profiles form complex cross-regulatory interactions [2], [3], [4], [5], [6]. In certain cases a cell population dependent upon a single bHLH gene can be replenished through a change in the fate of another population dependent on a different bHLH gene, as observed in spinal dorsal root ganglia development [7]. Transgenic misexpression of one bHLH gene under the promoter control of another bHLH gene results in diverse phenotypic outcomes depending on the tissue and the gene replaced [8], [9], [10]. In the retina, the bHLH gene *Neurod1* is needed for differentiation of amacrine cells and *Atoh7* for differentiation of retinal ganglion neurons [5]. However, misexpressing *Neurod1* under *Atoh7* promoter control rescues developing ganglion neurons [11]. This indicates a switch in context specific action of this misexpressed bHLH gene [8], possibly related to a sophisticated bHLH gene cross-regulation [4], [12] that may differ in the targeted tissue [11] or during certain developmental steps [13]. This variability of one bHLH gene to functionally replace another seems to relate in part to the similarities in the DNA binding domains, i.e., the E-boxes [14] and the complexity of the *cis* enhancer elements [3] for the different bHLH genes, but may also relate to the availability and type of the E-box associated protein binding partners [15], [16].

The inner ear is simpler developing system compared to the retina or the brain. The ear develops just two neurosensory cell types, hair cells for mechanotransduction and sensory neurons to conduct the information from the ear to the brain. Two bHLH transcription factors, *Atoh1* (formerly *Math1*) and *Neurog1* (formerly *Ngn1*) are necessary for neurosensory development in the inner ear. Eliminating either *Atoh1* or *Neurog1* leads to the absence of differentiated hair cells or neuron development in the mouse, respectively [17], [18]. In addition, several other bHLH genes [14] are also expressed in the inner ear and provide the molecular basis for the heterogeneity of a given neurosensory cell type [19]. While many cells in the inner ear will undergo apoptosis in the absence of their specific required bHLH gene, [20], [21], [22], under certain circumstances a transformation of one cell type into another cell type has been reported [23], [24], [25]. For example, in *Neurod1* conditional knockout mutants some cells in inner ear ganglia can differentiate as hair cells [19] through upregulation of *Atoh1* that is normally suppressed by *Neurod1.* These knockout data raise the possibility that other inner ear neurosensory cells could also react plastically when one bHLH gene is replaced by another through the altered cross-regulation of bHLH genes. Given that absence of *Neurog1* affects *Atoh1* mediated hair cell differentiation [23], we wanted to test the potential of fate changes for hair cell precursors to differentiate as neurons when *Atoh1* was replaced with *Neurog1* under *Atoh1* promoter control. To achieve this, we generated a knockin (KI) mouse where heterozygous KI mice (*Atoh1^+/KINeurog1^*) allowed us to assess the effect of co-expression of two different bHLH genes on hair cell development. We also bred these KI mice to homozygosity (*Atoh1^KINeurog1/KINeurog1^)* to test whether *Neurog1* could functionally replace *Atoh1* by either initiating differentiation of hair cell precursors or altering the fate of these precursors.

Our data show that *Neurog1* is expressed in hair cells of heterozygous KI mice and in clusters of undifferentiated organ of Corti precursors cells of homozygous KI mice where it regulates expression of *Neurod1* and several other hair cell-associated genes. In homozygous KI mice the patches of organ of Corti-like cells form microvilli and preserve afferent and efferent innervation instead of transforming into a ‘flat epithelium’ without any innervation as observed in *Atoh1* null or conditional knockout mice [17], [22]. We also show subtle but compelling evidence in heterozygous KI mice in altering the fate and patterning of both hair cell and supporting cells with gradual increasing severity of the defect with age. The phenotypes in the heterozygous KI mice indicate that *Atoh1* and *Neurog1* co-expression influence the extent of the morphological and histological defects. The data in homozygous KI mice suggest that replacement of *Atoh1* by *Neurog1* cannot fully rescue hair cell differentiation but can provide functional support for limited survival of organ of Corti-like patches without altering their fate to differentiate into neurons.

## Materials and Methods

All animal procedures were approved by the University of Iowa Institutional Animal Care and Use Committee (IACUC) guidelines for use of laboratory animals in biological research (ACURF #1103057).

### Generation of knockin (KI) mouse model (*Atoh1^KINeurog1^*)

#### Plasmids used

Six plasmid clones were utilized for the construction of the KI targeting vector. The pCS2-MT-Ngn1 plasmid was kindly provided by Dr. Qiufu Ma. This plasmid contains the full-length coding region of mouse *Neurog1* that was cloned into the pCS2-MT [26] vector from its original cDNA Bluescript plasmid (pBS) [27]. Both the pPGKneo-II (GenBank ID:AF335420) [28] and the PGKdtabpA [29] plasmids were obtained from the University of Nebraska Medical Center Mouse Genome Engineering Core Facility, Omaha, NE. pIRES2-DsRed2 was purchased from Clonetech (Mountain View, CA). Two pBSII KS clones containing genomic fragments isolated from a mouse 129/SvEv genomic DNA library (Stratagene) were kindly made available by Dr. Huda Zoghbi [30]. The pMath1-5′-9 plasmid contained an EcoRI/ApaI ∼10.5 kb fragment of the *Atoh1* locus. This fragment contains the 5′ flanking fragment (5.06 kb), the *Atoh1* open reading frame (∼1.06 kb) and a 4.38 kb 3′ flanking sequence. The second plasmid, pApa4.2, contains a 4.54 kb ApaI fragment that represents ∼164 bp of the 3′ end of the *Atoh1* coding sequence and a 4.38 kb sequence of the downstream 3′ fragment.

#### Construction of the Atoh1^KINeurog1^ plasmid

An initial plasmid was produced where a 1.29 kb IRES2-DsRed2 sequence was inserted 3′ to the 6XMyc-mNeurog1 sequence in the pCS2-MT-Ngn1 plasmid. The single *NotI* site in the original pIRES2-DsRed2 plasmid was replaced with a *XbaI* restriction enzyme recognition site. This was done by linearizing the plasmid by NotI enzymatic digestion, followed by blunt-ending using T4 polymerase. A XbaI adapter was then ligated with T4 DNA ligase onto the linearized plasmid and sticky ends were obtained through subsequent XbaI digestion. This modified plasmid was circularized by ligation with T4 DNA ligase. The resulting plasmid was then digested with BamHI, blunt-ended with T4 DNA polyermase and then digested with XbaI. Subsequently a 1.29 kb fragment containing the IRES2-DsRed2 sequence was gel purified. The pCS2-MT-Ngn1 plasmid was prepared for insertion of the IRES2-DsRed2 fragment by linearizing through XhoI digest, then blunt-ended with T4 DNA polymerase, followed by XbaI digestion, and then dephosphorylated using shrimp alkaline phosphatase. After gel purification this linearized plasmid was ligated with the blunt/sticky-ended IRES2-DsRed2 fragment to generate a pCS2-MT-6xMyc-mNeurog1-IRES2-DsRed2 plasmid.

The next step involved the insertion of the 5′ Atoh1 genomic fragment into the pCS2-MT-6xMyc-mNeurog1-IRES2-DsRed2 plasmid. A unique single *EcoRV* site was created in pCS2-MT-6xMyc-mNeurog1-IRES2-DsRed2 plasmid by partial DraI digestion to yield a linearized plasmid with only one of the four *DraI* sites (nucleotide positions 93, 3817, 3836 and 4528) being cut. *EcoRV* adapters were next ligated onto the ends of the linearized plasmids, followed by digested with EcoRV and then circularized by ligation. A clone containing an *EcoRV* site at position 93 was selected for further cloning steps. A linearized plasmid with sticky/blunt ends was produced by a double digestion using the ClaI and EcoRV restriction enzymes, which was then dephosphorylated with shrimp alkaline phosphatase. The *ClaI* site was immediately upstream of *EcoRV* site. The pMath1-5′-9 plasmid was initially digested with SphI and blunted with T4 DNA polymerase, which was then followed by complete digestion with ClaI to create *ClaI* sticky ends. The 5.07 kb fragment, which included 15 bps of the *Atoh1* coding sequence, was ligated into pCS2-MT-6xMyc-mNeurog1-IRES2-DsRed2 linearized plasmid to create a pCS2-MT-5′Atoh1-6xMyc-mNeurog1-IRES2-DsRed2 plasmid.

A plasmid, pBSII-loxP-pGKneo-3′Atoh1, was constructed that contained the floxed PKGneo cassette in reverse orientation to the 3′ *Atoh1* sequence. The pGKneo-II plasmid was partially digested with XbaI and blunted by T4 DNA polymerase treatment. The 4.77 kb linearized pGKneo-II plasmid was then digest with BamHI and treated with shrimp alkaline phosphatase. A 4.60 kb fragment containing the 3′ *Atoh1* sequence was generated by a partial digestion with KpnI to linearize the pApa4.2 fragment, which was then blunted by T4 DNA polymerase. This product was then digested with BamHI and the fragment was then gel purified, followed by ligation into the dephosphorylated linearized pGKneoII plasmid. The multilinker sequence in this construct was removed by partial digestion with SalI to yield a linearized plasmid and then digested with BamHI. The overhanging ends were blunted using T4 DNA ligase to generate the pBSII-loxP-PGKneo-3′Atoh1 plasmid.

The final knockin (KI) construct was created by linearizing the pBSII-loxP-PGKneo-3′Atoh1 through digestion of the adjacent *ClaI* and *EcoRV* sites with both their respective enzymes to create a sticky/blunt linearized plasmid. This DNA was then dephosphorylated with shrimp alkaline phosphatase. A 7.63 kb fragment containing the 5′Atoh1-6XMyc-mNeurog1-IRES2-DsRed2 sequence was prepared for insert into the prepared pBSII-loxP-PGKneo-3′Atoh1 dephosphorylated plasmid. The pCS2-MT-5′Atoh1-6XMyc-mNeurog1-IRES2-DsRed2 plasmid was linearized by NotI digestion and then treated with T4 DNA polymerase to create blunt ends, followed by digestion with ClaI. The resulting 7.63 kb fragment was then ligated into the pBSII-loxP-PGKneo-3′Atoh1 to generate a pBSII-5′Atoh1-6XMyc-mNeurog1-IRES2-DsRed2- loxP-PKGneo-3′Atoh1 plasmid and was designated as pAtoh1^KINeurog1^.

The Atoh1^KINeurog1^ sequence was then cloned into PGKdtabpA. The Atoh1^KINeurog1^ insert was prepared by digestion of pAtoh1^KINeurog1^ with ClaI followed by the blunting using T4 DNA polymerase and the desired fragment was excised with NotI digestion. This fragment was gel purified. The PGKdtabpA vector was prepared by partial SpeI digestion to linearize the plasmid, blunting with T4 DNA polymerase, followed by NotI digestion and then dephosphorylated by shrimp alkaline phosphatase. The Atoh1^KINeurog1^ sequence was then ligated with T4 DNA ligase into the gel purified linearized PGKdtabpA plasmid. The final pPGKdtabpA-Atoh1^KINeurog1^ plasmid was linearized by NotI digestion and gel purified. This fragment was used for transformation of the embryonic stem cells. All plasmid constructs were analyzed by restriction enzyme digestions and DNA sequence analyses of the ligated ends to confirm that the correct plasmids were generated. A complete sequence analysis of the Neurog1 insert was done to verify that no changes had occurred during the production of the pPGKdtabpA-Atoh1^KINeurog1^ plasmid.

#### Atoh1^KINeurog1^ production

Two 129/SvJ ES cell lines, E14 and R1, were used for targeting [31], [32]. The linearized pGKdtabpa-KI construct was injected into ES cell lines. The number of ES cell DNA after positive (Neo) and negative (HSV thymidine kinase and *Diphtheria* toxin) selection protocols were a total of 273 (103 derived from E14 and 170 derived from R1 ES cell lines). Genomic DNA samples were isolated from each clone and EcoRI digestions were done to initially identify those clones with putative homologous recombination. The wild-type genomic DNA produced a restriction fragment length (RFL) of 13.3 kb for both the 5′ and 3′ genomic probes. The predicted genomic RFL were 5.2 kb and 9.7 kb that was detected by the 5′ and 3′ probes, respectively. We were able to identify 48 ES cultures with EcoRI fragments of 5.2, 9.7 and 13.3 kb. DNA samples from 40 of these positive ES clones underwent HindIII digestions. We were able to identify 15 clones that demonstrated the predicted RFLs of 5.4 kb 5′ fragment and two 3′ RFLs, 19.5 kb and 22 kb, that represented the wild-type (*Atoh1* ^+^) and knockin (*Atoh1^KINeurog1^*; also referred to as *Atoh1^tm1Bfri^*) alleles, respectively. These ES cell lines were examined for their morphology and growth characteristics and five of those that demonstrated similar histological features to the original ES cell lines were selected for implantation into estrogen-primed female mice and was done by the University of Nebraska Medical Center Mouse Genome Engineering Facility using established procedures [31], [32]. Only the offspring from the 8C1 culture demonstrated germ line transmission of the *Atoh1^KINeurog1^* allele and thus produced the *Atoh1^KINeurog1^* mouse line.

#### Southern blotting

Southern blotting was done as previously described with some modifications [33]. Briefly, 5 μg of genomic DNA from each ES cell culture line was digested overnight at 37°C using 15 U of restriction enzyme per μg of genomic DNA. High fidelity EcoRI or HindIII enzymes (New England Biolabs, Ipswich, MA) were used. Digested DNAs were electrophoresed overnight on a 0.8% agarose gel. The gel was treated by soaking in a 0.25 N HCl solution, then the DNA was denatured using 0.5 M NaOH solution, followed by a 10X SSC solution (1.5 M NaCl/150 mM sodium citrate, pH 7.0) and then blotted onto Hybond-N^+^ filters (Amersham, Piscataway, NJ) using a vacuum blotter system (BioRad, Hercules, CA) following the manufacturer's instructions. Probes were amplified by two primer sets specific for genomic DNA up- and downstream of the mouse *Atoh1* gene. 5′ probe: gmAtoh1 5′-FOR (5′ CTGAGGAATACCGAATGGCAGAG 3′) and gmAtoh1 5′-REV (5′ CTACTTCCCTAACCACCCATTCC 3′) with an 1102 bp amplicon.

3′ probe: gmAtoh1 3′-FOR (5′ CATGCTGACTGG TTCCTTTCTCTC 3′) and gmAtoh1 3′-REV (5′ GGTCTGGCTTCDTGTAAACTCTGC 3′) that yielded 1195 bp product. Purified PCR amplicons were then random primer labeled using 6000 Ci/mml dCTP (α^32^P) and dATP (α^32^P) as per manufacturer's protocol (Roche Applied Science, Indianapolis, IN). Filters were hybridized overnight at 65°C [33] and washed using high stringent conditions (0.1X SSC, 1% SDS) at 65°C. The filters were developed using a Storm Phosphorimager and the resulting images analyzed to determine the number and sizes of the detected RFLs.

#### Genotyping

Genotyping of *Atoh1^KINeurog1^* mice was completed using tail DNA for standard PCR amplification. The PCR conditions consisted of an initial denaturing step at 94°C for 2 minutes, an amplification step for 32 cycles at 94°C for 30 seconds, 55°C for 30 seconds, and 72°C for 1 minute, and a final elongation step at 72°C for 10 minutes. EconoTaq plus green 2X master mix (Lucigen, 30033) and a three primer set were used for these reactions. All resultant products were electrophoresed and visualized on a 2% agarose gel. The three primer set consisted of KI-PKG-9225 (3′ forward) with a sequence 5′-CTA CCC GCT TCC ATT GCT CAG C-3′, GT gmAtoh1-9179 (3′ reverse) with a sequence of 5′-ACT CTC CGT CAC TTC TGT GGG ATC-3′, and Atoh1 S1 with a sequence of 5′-GAC CAC CAT CAC CTT CGC ACC-3′. The wild-type *Atoh1* allele (*Atoh1^+^*) was determined by the primer set Atoh1 S1 and GT gm Atoh1-9179 and produced a ∼300 bp product. The KINeurog1 allele was detected with the primer set KI-PKG-9225 and GT gmAtoh1-9179 and produced a ∼600 bp product. *Atoh1* conditional knockout (CKO, *Pax2-cre; Atoh1 ^f/f^*) mice were generated by crossing the floxed *Atoh1* with the *Tg* (*Pax2-cre*) line described previously [17], [22].

### *In situ* hybridization

*In situ* hybridization was performed using the RNA probe labeled with digoxigenin. The plasmids containing the cDNAs were used to generate the RNA probe by *in vitro* transcription. Locked nucleic acid (LNA) probes for microRNAs (*miR-96 and miR-124*) were purchased and used as described previously (miRCURY LNA probes; Exiqon, Woburn, MA; [34]). *Ntf3* antisense probe was made using the IMAGE clone 1177923 and EcoRI restriction enzymes and T3 RNA polymerase was used. After being anesthetized with Avertin, mice were perfused in 4% paraformaldehyde (PFA) and fixed overnight in 4% PFA. The ears were dissected in 0.4% PFA and dehydrated and rehydrated in graded methanol series and then digested briefly with 20 µg/ml of Proteinase K (Ambion, Austin, TX, USA) for 15-20 minutes. Then the samples were hybridized overnight at 60°C to the riboprobe in hybridization solution containing 50% (v/v) formamide, 50% (v/v) 2X saline sodium citrate (Roche) and 6% (w/v) dextran sulphate. After washing off the unbound probe, the samples were incubated overnight with an anti-digoxigenin antibody (Roche Diagnostics GmbH, Mannheim, Germany) conjugated with alkaline phosphatase. After a series of washes, the samples were reacted with nitroblue phosphate/5-bromo, 4-chloro, 3-indolil phosphate (BM purple substrate, Roche Diagnostics, Germany) which is enzymatically converted to a purple colored product. The ears were mounted flat in glycerol and viewed in a Nikon Eclipse 800 microscope using differential interference contrast microscopy and images were captured with Metamorph software. The ears of the littermate of different genotype for the same gene expression were performed in the same reaction tubes to maintain the reaction accuracy.

### Immunochemistry

The mice for immunofluorescent staining were collected as mentioned previously and fixed in 4% PFA overnight, the ears were dissected and dehydrated in 100% ethanol and then rehydrated in graded ethanol series and in PBS and blocked with 2.5% normal goat serum in PBS containing 0.5% Triton-X-100 for 1 hour. Then the primary antibodies for Myo7a (Myosin 7a, Proteus Biosciences), Tubulin (Sigma), Sox2 (Millipore), activated Caspase3 (Cell Signaling Technology), DsRed2 (anti-red fluorescent protein; Genway) and Prox1 (Covance) were used in dilutions of 1∶200, 1∶800, 1∶200, 1∶100, 1∶1000 and 1∶200, respectively. and incubated for 24-48 hours at 4°C. After several washes with PBS, corresponding secondary antibodies (1∶500) (Alexa fluor molecular probe 647 or 532 or 488; Invitrogen) were added and incubated overnight at 4°C. Hoechst nuclear stain (Polysciences; 10mg/ml) was used at room temperature for 1 hour. The ears were washed with PBS and mounted in glycerol and images were taken with a Leica TCS SP5 confocal microscope.

### Scanning electron microscopy (SEM)

The mice for scanning electron microscopy were perfused and fixed in 2.5% gluteraldehyde in 1% PFA after sedating with Avertin. Ears of postnatal mice were decalcified with EDTA. Following osmication in 2% osmium tetroxide in 0.1 M phosphate buffer (pH 7.4) for up to 1 hour, the ears were microdissected including removal of the Reissners membrane and the tectorial membrane. The samples were then washed several times with distilled water to remove ions, dehydrated in a graded ethanol series, critical point dried, mounted on stubs and coated with gold/palladium. Stubs were then viewed with a Hitachi S-4800 Scanning Electron Microscope with 3MeV acceleration.

### Plastic embedding and Stevenel's Blue staining

The ears for plastic embedding were fixed in 2.5% gluteraldehyde in 1% PFA, washed in 0.1 M phosphate buffer, dehydrated in ethanol series and in propylene oxide. The samples were then embedded in Epon 812 in beam capsules and incubated for 24–48 hours in 60°C oven. Two µm thin sections were cut with a Leica RM2265 Microtome using a diamond knife. Some sections were stained with Stevenel's Blue as described previously [19] for detailed histology.

### Dye Tracing

After perfusion with 4% PFA, the heads of mice were hemisected and lipophilic dye-soaked filter strips (Fritzsch, 2005) were inserted into the basal plate of rhombomere 4 for labeling of the efferent fibers to the inner ear. After the injection of dye into the brains, the hemisected heads were incubated at 60°C for 3 days for proper diffusion. The inner ears were then dissected out, mounted on glass slides in glycerol, and imaged immediately with a Leica TCS SP5 confocal microscope.

### Western Blot Analysis

P5 and P9 heterozygous mice were decapitated after a lethal dose of Avertin and brains (cerebella) of the mice were snap frozen in liquid nitrogen and stored at -80°C. Then the cerebella were homogenized in radio immune precipitation assay (RIPA) buffer with 1% protease inhibitor cocktail (Roche, Germany) and was clarified by centrifugation at 13,000 rpm for 15 min at 4°C. Protein concentration in the brain lysate was measured using a Pierce BCA protein assay kit (Thermo scientific, Rockford, IL), protein was denatured at 95°C for 5 minutes and 30 µg of protein samples were then loaded in a 10% polyacrylamide gel. The samples were then blotted onto nitrocellulose membranes and probed with the primary antibodies of Neurog1 (Neurogenin1; 1∶ 500; Abcam, ab89461), Myc-tag (1∶500; Millipore; clone 9E10-05-419) and β-catenin (1∶500; BD Transduction Laboratories) overnight at 4°C. Species specific HRP secondary antibodies (1∶ 5000; Thermo scientific, Rockford, IL) were used for 1 hour at room temperature. The protein bands were identified using Chemiluminescent substrates (Thermo scientific, Rockford, IL).

## Results

### *Atoh1^+/KINeurog1^* mice were viable and showed co-expression of *Atoh1* and Neurog1 in the same cells whereas *Atoh1^KINeurog1/KINeurog1^* mice were early lethal

The heterozygous KI (*Atoh1^+/KINeurog1^)* mice were viable and displayed no obvious behavioral abnormality. We crossed the heterozygous mouse to generate a homozygous KI mouse (*Atoh1^KINeurog1/KINeurog1^*) in which *Atoh1* was replaced by *Neurog1* in both alleles. At embryonic day 13.5 (E13.5), E14.5, E16.5 and E18.5 mice with the genotypes of *Atoh1^+/+^*, *Atoh1^+/KINeurog1^* and *Atoh1^KINeurog1^* ^/*KINeurog1*^ were obtained in a nearly 1∶2∶1 ratio. Homozygous KI mice were infrequently found within a few hours after birth, but were never present in litters 24 hours or older. These suggested that the *Atoh1^KINeurog1^* ^/*KINeurog1*^ mice were lethal within a few hours after birth, much like *Atoh1* null mice [17]. We therefore concentrated our analysis on embryonic stages (E14.5, E16.5 and E18.5) and newborn (postnatal P0 animals) in homozygous KI mice and embryonic as well as several postnatal stages (P1, P7, P9, P26) in heterozygous KI mice to investigate the degree of differentiation of hair cells when *Atoh1* was partially or completely replaced by *Neurog1*. In the rest of the text we will refer to the *Pax2-cre; Atoh1 ^f/f^* mice as *Atoh1* CKO (conditional knockout mice) and knockout *Atoh1^-/-^* as *Atoh1* null.

**Figure 1:**
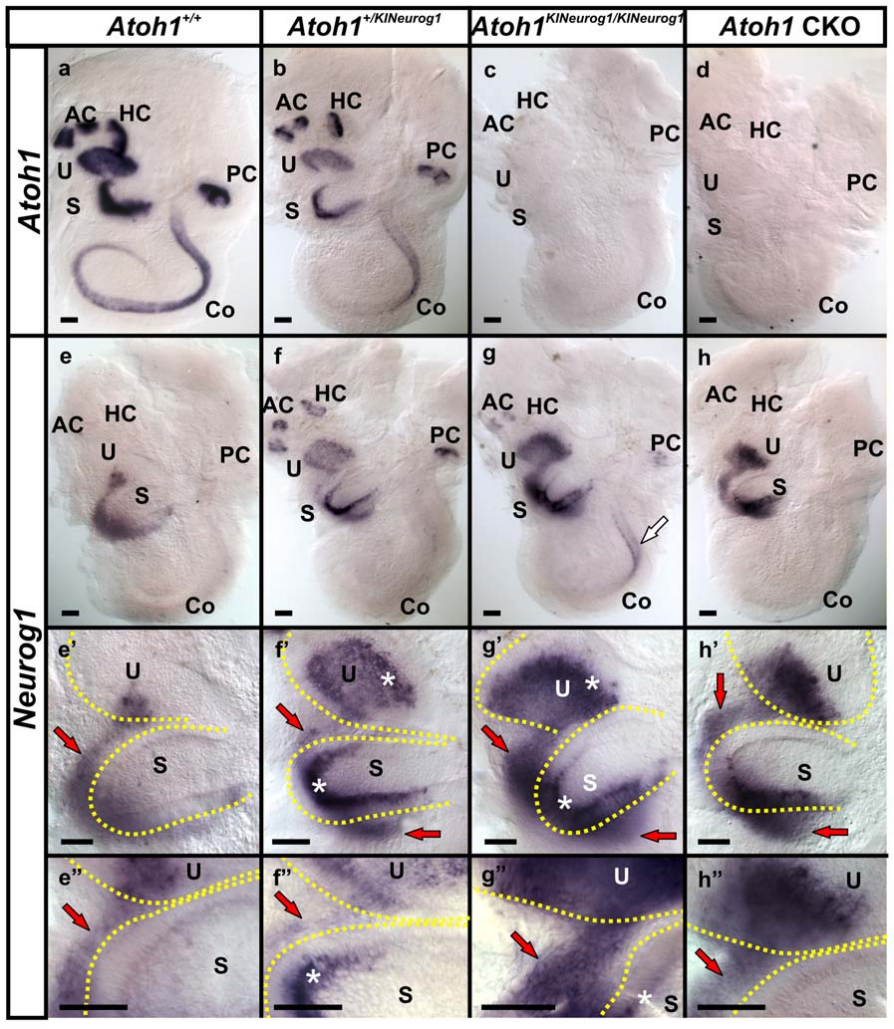
Replacement of *Atoh1* with *Neurog1* mimics spatio-temporal expression of *Atoh1*. *In situ* hybridization at E14.5 shows the expression of *Atoh1* in a wild-type (*Atoh1^+/+^*) mouse (a) which is less profound in the heterozygous KI (*Atoh1^+/KINeurog1^*) littermate (b). There is no *in situ* signal for *Atoh1* mRNA in the homozygous KI (*Atoh1^KINeurog1/KINeurog1^*) (c) and in the *Atoh1* conditional knockout mice (CKO, *Pax2cre; Atoh1^f/f^*) (d). *Neurog1 in situ* hybridization in the wild-type control mice represents expression only in the delaminating neurons emanating from the utricle and saccule but not in the sensory epithelia (e, e’,e”). In heterozygous KI mice, *Neurog1* is expressed in the vestibular sensory epithelia, but not yet in the cochlea and is diminished in the delaminating neuroblasts near the utricle and saccule compared to the wild-type littermate (compare red arrows in e, e’,e” and f, f’, f”). Both homozygous KI and *Atoh1* CKO mice show expression of *Neurog1* in the delaminating neuroblasts and in the epithelia of the utricle and saccule (g, g’, g”, h, h’,h”). In addition, *Neurog1* is expressed in the canal cristae and the mid-base of the cochlea of homozygous KI mice (white arrow in g) which is not observed in the *Atoh1* CKO cochlea (h). The expression of *Neurog1* in the sensory epithelia recapitulates *Atoh1* expression (compare a, g) whereas the more obvious expression of *Neurog1* in delaminating neurons is observed in *Atoh1* CKO mice (h). Co-expression of *Atoh1* with *Neurog1* in the developing hair cells restricts *Neurog1* expression in delaminating neurons in the heterozygous KI mice below the level encountered in wild-type, homozygous KI and *Atoh1* CKO mice (Red arrows indicate the delaminating neuroblasts; asterisks indicate the sensory epithelia; yellow dotted lines demarcate the boundaries of the utricle and saccule in e’,e”, f’, f”, g’, g”, h’,h”). AC, anterior crista; Co, Cochlea; HC, horizontal crista; PC, posterior crista; S, saccule; U, utricle. Bar indicates 100 µm.

*In situ* hybridization in E14.5 mice showed no detectable *Atoh1* expression in homozygous KI, similar to *Atoh1* CKO in the ear (Fig. 1c,d). Heterozygous KI mice showed delayed and/or downregulated *Atoh1* expression in the cochlea (Fig. 1a, b). These mice also showed expression of *Neurog1* in the vestibular hair cells. In contrast, wild-type mice displayed *Neurog1* expression only in the delaminating neuroblasts. Endogenous *Neurog1* expression was reduced in the delaminating neuroblasts in heterozygous KI mice compared to wild-type littermate (Fig. 1e–e”, f-f”). This indicates that the co-expression of *Atoh1* and *Neurog1* in the hair cells influence delaminating neuroblast expression of *Neurog1* (Fig. 1f–f”), possibly by altering the previously established cross-regulation [24]. In homozygous KI mice, *Neurog1* was expressed in all vestibular sensory epithelia and in the mid-base region of the cochlea at E14.5 (Fig. 1g), following the usual expression pattern of *Atoh1*. Homozygous KI mice differed from *Atoh1* CKO mice which showed no expression of *Neurog1* in either canal cristae or cochlea (Fig. 1h). However, homozygous KI mice showed expansion of *Neurog1* expression in the delaminating neuroblasts like *Atoh1* CKO mice (Fig. 1g–g”, h-h” and as reported previously [22], [24]). These data show that the KI allele with *Neurog1* being driven by the native *Atoh1* promoter (Fig. S1) is expressed as intended and permits misexpression of *Neurog1* in the inner ear hair cells as well as altering the expression patterns of nearby cells.

**Figure 2:**
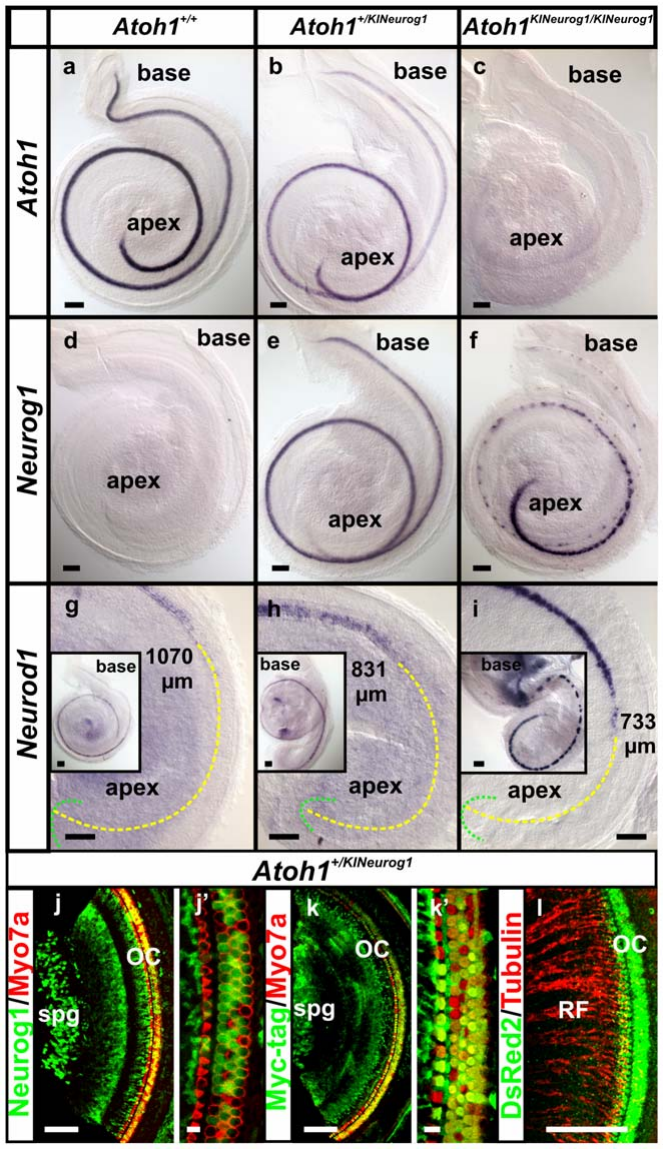
*Neurog1* and *Neurod1* expression is retained in the organ of Corti cells in the KI mice that have no *Atoh1* expression. *Atoh1 in situ* hybridization shows a reduced expression in E18.5 heterozygous KI mice compared to the wild-type littermate (a, b) but complete absence in homozygous KI mice (c). *Neurog1* is absent in the wild-type cochlea by E18.5 (d) but present in the organ of Corti of the heterozygous KI cochlea (e). Homozygous KI mice show a strong expression of *Neurog1* in discontinuous cluster of cells in the organ of Corti, except for a continuous expression in the apex of the cochlea (f). *Neurod1*, a downstream gene to *Neurog1* and *Atoh1* is relatively more advanced toward the apex in the cochlea of heterozygous KI mice than the wild-type littermate (inserts in g, h; compare the distance in g,h). In homozygous KI mice *Neurod1* is strongly expressed in discontinuous patches of organ of Corti cells and some spiral neurons (i) and almost reaches the apical tip (compare g, h, i). The distance measured from the apical tip of the cochlea to the *Neurod1* expression is marked with yellow dotted lines (g,h,i). Immunochemistry in E18.5 heterozygous KI mice demonstrates both Neurog1 and Myc-tag immunopositivity in the hair cells as well as in the spiral ganglia (j-k’). Neurog1 and Myc-tag immunopositivity in hair cells is shown together with Myo7a, a marker for hair cells (j-k’). DsRed2, a protein marker for *Neurog1* expression showed localization only in the hair cells in the heterozygous KI mice which are innervated by the radial fibers labeled with anti-Tubulin antibody (l). OC, organ of Corti; RF, radial fibers; Spg, spiral ganglia. Bar indicates 100 µm except j’ and k’ where it indicates 10 µm.

The normal spatio-temporal initiation of *Neurog1* expression in *Atoh1*-expressing hair cell precursors at E14.5 could potentially lead to normal hair cell differentiation (Fig. 1g). In contrast to this expectation, *Neurog1* was expressed in E18.5 homozygous KI mice only in clusters of organ of Corti cells, except for continuous expression in the apical tip demonstrated by *in situ* hybridization (Fig. 2f). *Atoh1* expression in the homozygous KI mice was eliminated as expected but was also reduced in the heterozygous KI cochlea, specifically in the basal half compared to the wild-type littermate (Fig. 2a, b, c). *Neurog1* and *Atoh1* were expressed in hair cells of E18.5 heterozygous mice (Fig. 2a,b,d,e). *Neurod1*, an immediate downstream gene of *Neurog1* [18], was expressed in both wild-type and in heterozygous cochlea at E18.5 (Fig. inserts in 2g and h). *Neurod1 in situ* showed a similar patchy distribution in the homozygous KI cochlea as observed by *Neurog1* (Fig. inserts in 2i). *Neurod1* upregulation was earlier in the apex of E18.5 homozygous and heterozygous KI mice than wild-type littermate (Fig. 2g–i). *Neurod1* was also prematurely expressed at E16.5 in heterozygous cochlear hair cells compared to wild-type littermate where it was only expressed in neurons (data not shown).

6X Myc-tag epitope and DsRed2, the fluorescent protein marker were added onto the engineered *Neurog1* to detect the Neurog1 protein expression. Immunochemistry for Neurog1, Myc-tag and DsRed2 showed distribution in the hair cells in E18.5 heterozygous KI cochlea (Fig. 2j–l) as well as some non-specific staining of Neurog1 and Myc-tag in the spiral ganglia (Fig. 2j, k). The localization of Neurog1 and Myc-tag protein in the hair cells was correlated by co-labeling with the hair cell marker, Myo7a, in the heterozygous KI mice (Fig. 2j–k’). DsRed2 was co-labeled with anti-Tubulin antibody showing the projection of radial fibers to these DsRed2- positive hair cells (Fig. 2l). In addition, we identified Neurog1 and Myc-tag proteins at the approximate expected size (∼25 kDa and ∼36 kDa, respectively) by western blot analysis using the cerebella of P5 and P9 heterozygous KI mice (Fig. S2a). However, the same sized protein bands for Neurog1 and Myc-tag were also found in the wild-type samples which suggested non-specific protein detection (Fig. S2b). Antibodies against several bHLH transcription factors are generally problematic, requiring novel approaches to overcome these problems [35].

Distribution of *Neurog1* in cells within patches of organ of Corti in homozygous KI mice indicated either a differential expression of *Neurog1* in hair cells precursors or loss of some hair cell precursors. *Neurog1* distribution in these mice resembled the discontinuous distribution of remaining *Atoh1^lacZ^* positive cells reported in E18.5 *Atoh1^lacZ/lacZ^* null mutants [36] but was more patchy and showed more rows of cells within the patches. Besides inner ear, misexpression of *Neurog1* revealed comparable replacement of *Atoh1* expression by *Neurog1* in brains as well where *Neurog1* recapitulated *Atoh1*-pattern in the homozygous KI mice both in the cochlear nucleus and in proliferating external granule cells of the cerebellum (Fig. S3c,c’,f,f’,f”).

### *Neurog1* misexpression in homozygous KI mice rescued clusters of organ of Corti precursors

**Figure 3:**
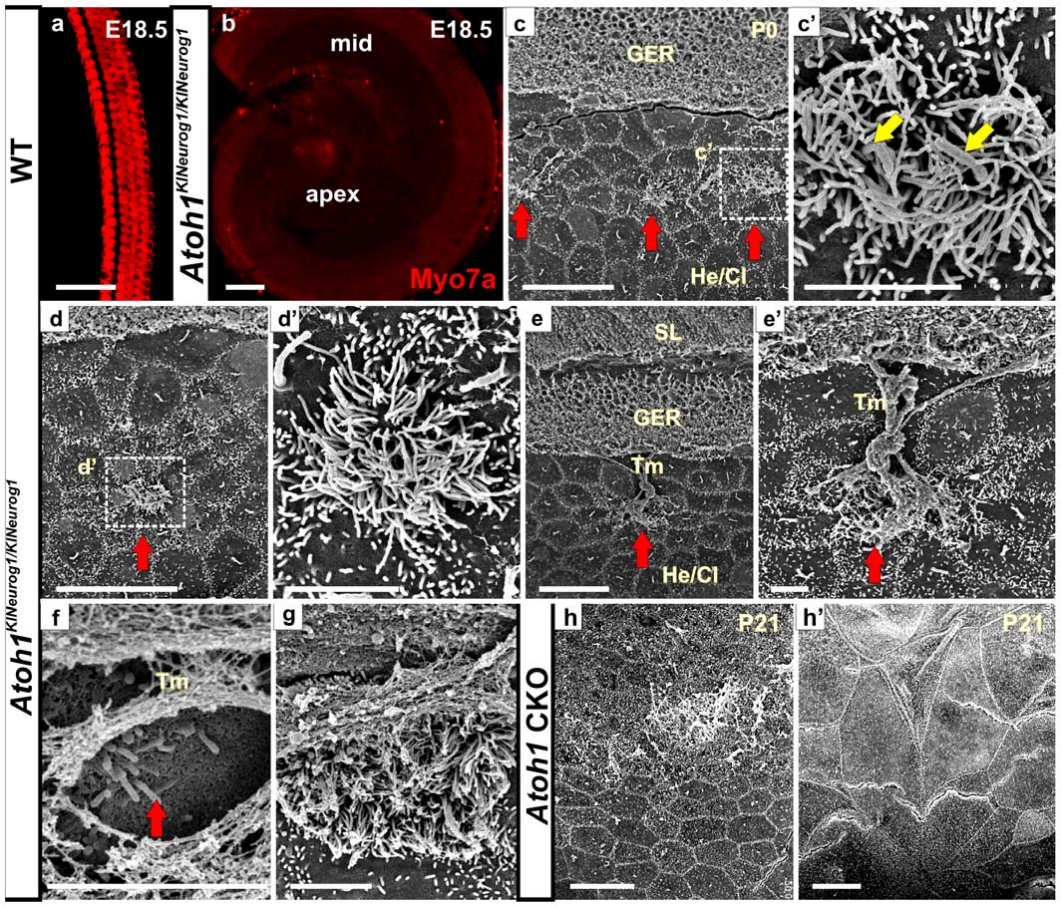
*Neurog1* misexpression results in survival of some organ of Corti cells in homozygous KI mice. Immunochemistry in E18.5 mice shows no Myo7a expressing cells in the organ of Cori of the homozygous KI mice compared to wild-type mice (a,b). Scanning electron microscopy in the P0 homozygous KI mice (c-g) display patches of organ of Corti cells with formation of microvilli or rudimentary stereocilia (red arrows in c, d, e, e’, f) surrounded by ‘flat epithelium’ composed of Claudius cell-like cells. Some of these cells are adjacent to each other with two central kinocilia (yellow arrows in c’). These clusters of cells are mostly in the position of outer hair cells and are surrounded by organized Claudius cell-like cells (c, d, e). The tectorial membrane is largely confined to the GER but extents selectively to these patches of microvilli bearing cells, many having attachment of protrusions of tectorial membrane on their surfaces (e-g). Lack of *Atoh1* in *Atoh1* CKO results in a continuous ‘flat epithelium’ without formation of any presumed sensory cells bearing microvilli (h,h’). GER, greater epithelia ridge; He/Cl; Hensen’s cells and Claudius cells; IHC, inner hair cells; OHC, outer hair cells; Tm, tectorial membrane; SL, spiral limbus. Bar indicates 100 µm in a,b; 10 µm in c, d, e, h, h’; and 2 µm in c’, d’, e’, f, g.

Homozygous KI mice had no detectable Myo7a expression (Fig. 3b) in comparison to the wild-type cochlea showing normal organization of Myo7a immunofluorescence labeled hair cells (Fig. 3a). To investigate the morphology of *Neurog1* positive cells within the organ of Corti-like patches and their associated ultrastructure in the homozygous KI mice, we examined newborn (P0) mice with scanning electron microscopy (SEM) (Fig. 3c–g). Previous reports on *Atoh1* null mice [17] and our observations on *Atoh1* CKO mice (Fig. 3h, h’; [22]) showed a uniform ‘flat epithelium’ that is not covered by a tectorial membrane. In contrast, we found formation of single or cluster of cells with long microvilli or rudimentary stereocilia without any staircase patterned stereocilia in homozygous KI mice (Fig. 3c–g). These organ of Corti cells were localized in patches (Fig. 3c–g) and in most cases were associated with an expansion of the tectorial membrane that was otherwise confined to the greater epithelial ridge (GER) (Fig. 3e–g). These cells were always in the center of Claudius-like cells (Fig. 3c, d, e) indicating some cellular interaction to organize cells around the cells bearing microvilli. Our data suggested that misexpression of *Neurog1* can rescue some organ of Corti cells to obtain some degree of differentiation (Fig. 3c–g) instead of forming a ‘flat epithelium’ as in the *Atoh1* CKO mice (Fig. 3h, h’).

### Co-expression of *Atoh1* and *Neurog1* in heterozygous KI mice resulted in progressive disorientation of hair cells and supporting cells including deviation of hair cell polarity

**Figure 4:**
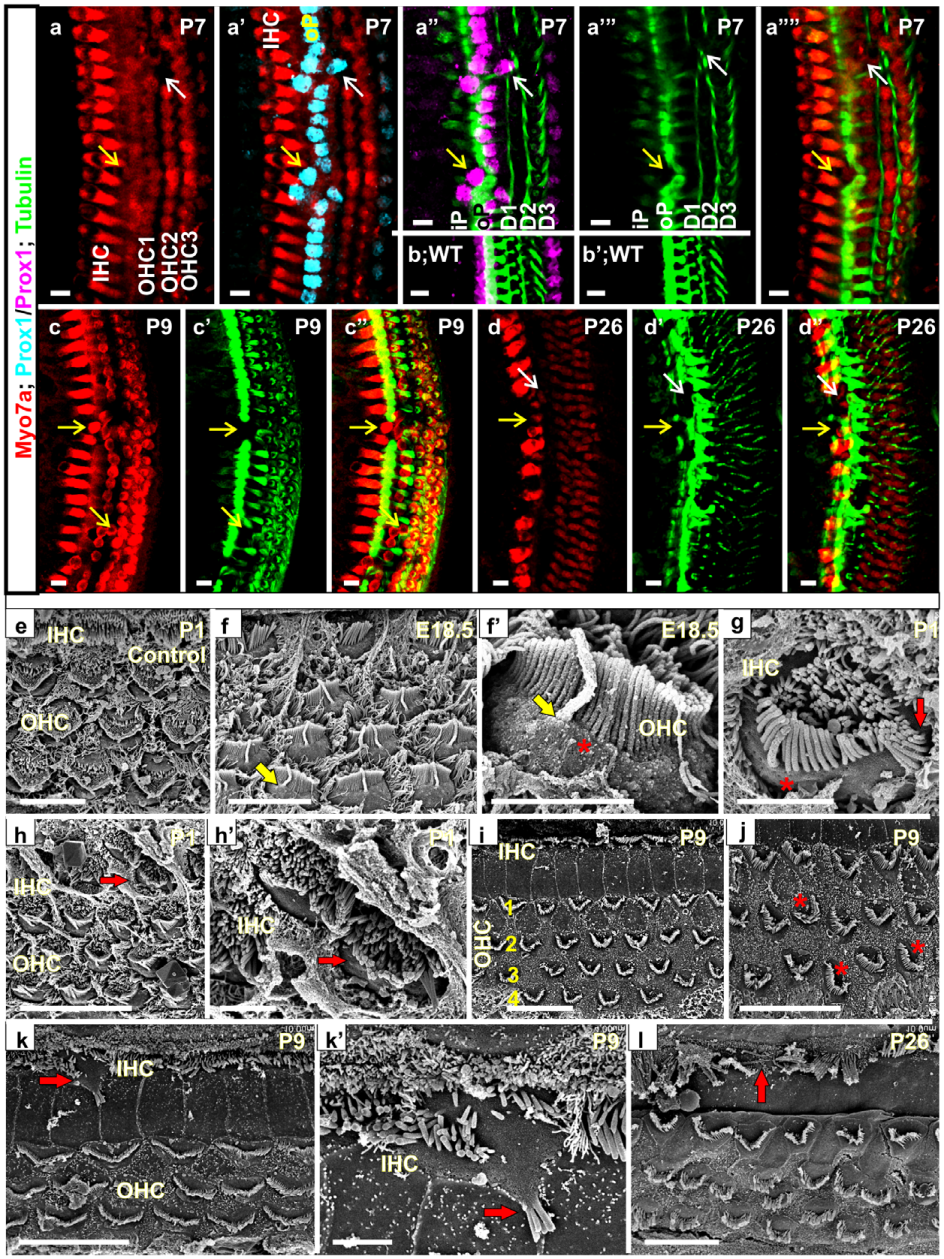
*Neurog1* expression causes disorganization of both hair cells and supporting cells in the heterozygous KI mice. Immunochemistry of Myo7a shows normal rows of hair cells with loss of some outer hair cells in P7 heterozygous KI mice (white arrows in a, a’, a””). Co-labeling of Myo7a with the supporting cell-specific markers, Prox1 and Tubulin shows the loss of few outer hair cells in areas of misaligned and/or ectopic pillar cell formation as well as disorganization of Deiters' cells (white arrows in a’-a””; b,b’). Some ectopic hair cells are found in the position of inner hair cells more apparently at P9 and also in between the rows of outer hair cells which are associated with the disorganization of supporting cells (yellow arrows in a- a””, c-c”) followed by loss of some inner hair cells and pillar cells by P26 (arrows in d-d”). Anti-Prox1 specifically labels outer pillar cells and third rows of Deiters’ cells at P7 whereas anti-Tubulin strongly labels outer pillar and all three rows of Deiters' cells. Anti-Tubulin more weakly labels inner pillar cells in heterozygous (a’-a””, c’,c”,d’,d”) and wild-type cochlea (b, b’). Prox1 is shown in cyan in a’ and lilac in a” and b for better contrast. Scanning electron microscopy P1 control (*Pax2-cre; Atoh1^+/f^*) mice reveal four parallel rows of hair cells in the organ of Corti with a staircase-pattern of stereocilia (e). SEM in E18.5 to P26 heterozygous KI mice reveals defects in hair cell stereocilia development and patterning (f-l). Hair cells show kinocilia that are not centered in relation to the symmetry of the stereocilia bundle (shown with yellow arrows and red asterisks in f,f’,g). At P1, some abnormal patches of stereocilia form in the inner hair cells (red arrow in g) as well as some ectopic hair cells adjacent to inner hair cells (red arrows in h and h’). At P9, occasionally extra rows of outer hair cells form with unequal length of the ‘W’ end of the stereocilia (i, red asterisk in j). In addition, the stereocilia of inner hair cells are sporadically disrupted or fused abnormally (k, k’) and some inner hair cells at P26 are missing (arrow in l) consistent with gaps seen with the Myo7a immunochemistry. Bar indicates 10 µm except 2 µm in e’, f’, g’, j’.

We next examined if the co-expression of *Neurog1* and *Atoh1* could affect hair cell differentiation. Myo7a and Tubulin immunochemistry indicated near normal hair cell differentiation with normal appearing organ of Corti cytoarchitecture at E18.5, except for some occasional misalignment of inner hair cells (data not shown). As these mice are viable, we analyzed later stages of development. At P7, immunochemistry of Myo7a revealed loss of some outer hair cells but near normal organization of inner hair cells (Fig. 4a). Disorganization became more apparent in supporting cells at this stage. Immunochemistry of Prox1 and Tubulin showed pillar and Deiters' cells were displaced in the rows of inner hair cells and/or outer hair cells which were associated with the loss of outer hair cells (Fig. 4a’-a””, b,b’). This disorganization in heterozygous KI mice was more obvious at P9 showing ectopic Myo7a positive outer hair cell-like cells in the rows of inner and outer hair cells including loss of some pillar cells (Fig. 4c–c”). The extent of the organ of Corti disruption appeared more at P26 and involved also in loss of some inner hair cells (Fig. 4d–d”). Closer investigation with SEM revealed further disorganization in hair cell polarity and stereocilia (Fig. 4f–l). These phenotypes in the heterozygous KI mice were compared with the *Pax2cre; Atoh1^f/+^* mice which confirmed the defect was not caused by the haploinsufficiency of *Atoh1* but rather the co-expression of *Neurog1* (Fig. 4e). Kinocilia have been implicated to act as a receptor for planar cell polarity signals to determine the correct polarity of the cell and the stereocilia [37], [38]. During development, kinocilia progressively move from the center of the bundle to the edge of the tall stereocilia providing the symmetry of the bundle [37], [38]. In heterozygous KI mice we found that in many hair cells, predominantly in the outer hair cells the stereocilia bundle was not arranged symmetrically in relation to the kinocilia (Fig. 4f, f’). At P1, occasionally inner hair cells were found with formation of additional stereocilia bundle (Fig. 4g) and formation of ectopic hair cells adjacent to the inner hair cells without formation of supporting cells in between (Fig. 4h,h’). At P9, the outer hair cell stereocilia asymmetry became more profound including formation of extra rows of outer hair cells in the cochlear apex (Fig. 4i, j), which was not observed in the base (Fig. 4k). In addition, we found occasional formation of ectopic stereocilia adjacent to inner hair cells at P9 (Fig. 4k,k’) resembling the formation of Myo7a positive ectopic hair cells in the gaps of pillar cells (Fig. 4a”’,a””,c–c”) as well as later loss of some inner hair cells at P26 (Fig. 4l, d,d”). The heterozygous KI mice affected the patterning of both hair cells and supporting cells suggesting cellular interaction with adjacent supporting cells.

### Expression of *Neurog1* could regulate downstream gene expression and influence patterning of the organ of Corti by differential regulation of some molecular markers

**Figure 5:**
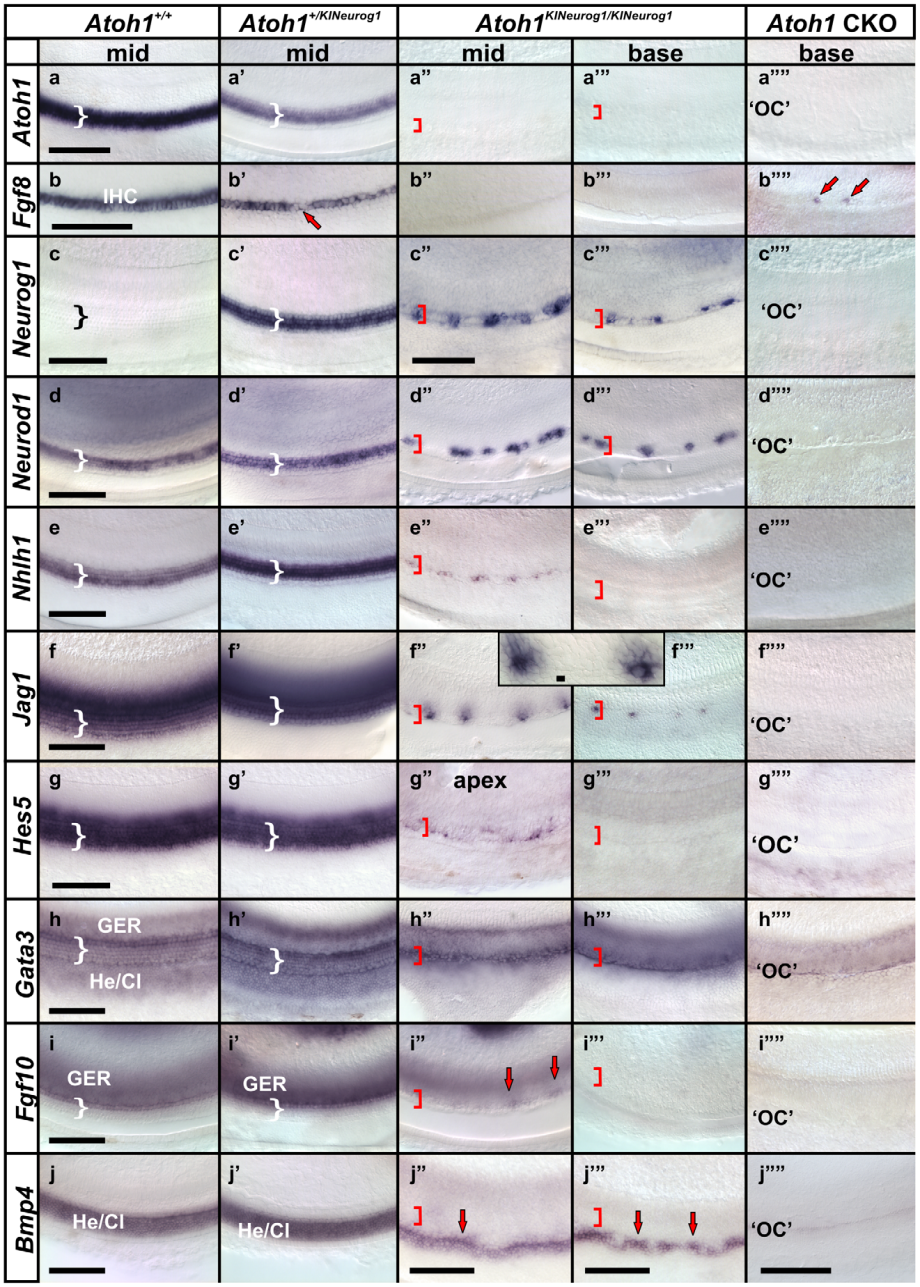
Expression of *Neurog1* in the organ of Corti affects expression of many genes in organ of Corti cells and in surrounding cells. These images of the comparable segments of E18.5 cochlea display expression differences of several genes in and around the organ of Corti for four different mouse genotypes. *In situ* hybridization of *Atoh1* shows a reduced expression in the heterozygous KI mice (a’) compared to wild-type littermates (a) and complete loss of *Atoh1* in the homozygous KI mice and *Atoh1* CKO mice (a”-a””). *Fgf8* is expressed only in inner hair cells in wild-type mice (b), shows reduction and loss in some inner hair cells (arrow in b’) in heterozygous KI mice, is absent in homozygous KI mice (b”,b”’) but is faintly expressed in some organ of Corti cells in the absence of *Atoh1* (arrow in b””). *Neurog1* is absent in the organ of Corti in wild-type mice (c) and *Atoh1* CKO mice (c””), but is present in the hair cells in heterozygous KI mice (c’) and shows a patchy distribution in the organ of Corti cells in the homozygous KI mice (c”, c”’). *Neurod1* and *Nhlh1* are downstream genes of *Neurog1* and *Atoh1* and are absent in *Atoh1* CKO mice (d””, e””) but present in wild-type and heterozygous KI mice (d,d’, e, e’). In the homozygous KI mice both genes are present in clusters of cells with progressive reduction towards the base (d”,d”’, e”, e”’). *Jag1*, a Notch ligand, is widely expressed in wild-type (f) and in heterozygous KI mice (f’), is expressed in patches of cells in the homozygous KI mice (f”, f”’) and is completely absent in *Atoh1* CKO mice (f””). Notch effector gene, *Hes5*, shows expression in the supporting cells and in GER both in wild-type and in heterozygous KI mice (g, g’). *Hes5* is massively diminished in the homozygous KI cochlea with some patchy retention only in the apex of the cochlea (g”, g”’) and is completely absent in the *Atoh1* CKO (g””). *Gata3* is expressed in a wide area in the cochlear epithelium in wild-type and heterozygous KI mice (h, h’), shows retention in the homozygous KI mice with a wavy appearance near the presumed organ of Corti (h”,h”’) and is much reduced in *Atoh1* CKO mice (h””) compared to the base of the homozygous KI cochlea (h”’). *Fgf10* and *Bmp4* are markers of the medial and lateral boundary of the organ of Corti (i, j) and are expressed almost identical in wild-type and heterozygous knockin mice (i, i’, j, j’). Expression of these markers are reduced in homozygous KI mice and show a wavy and discontinuous appearance (arrows in i”, j”, j”’), probably flanking the remaining patches of organ of Corti cells still expressing organ of Corti marker genes. Both *Bmp4* and *Fgf10* expressions are further reduced in the base of *Atoh1* CKO mice, reflecting the near complete loss of the organ of Corti in these mice (i””, j””). GER, greater epithelial ridge, He/Cl, Hensens’ and Claudius cells; IHC, inner hair cells, ‘OC’, putative organ of Corti in *Atoh1* CKO mice. ‘{‘ indicates the differentiated organ of Corti and ‘[‘ marks the presumed organ of Corti in the homozygous KI mice. Arrows indicate gaps in expression. Bar indicates 100 µm.

We next examined how the altered phenotype in organ of Corti cells was associated with the gene expression patterns in the E18.5 KI mice. Expression of *Neurog1* in the *Atoh1* locus not only provided *Neurog1* expression but also altered the expression of several inner ear specific genes in organ of Corti cells which differed from *Atoh1* CKO mice (Fig. 5). *Fgf8,* a gene selectively expressed in the inner hair cells, was reduced comparable to *Atoh1* expression (Fig. 5a, a’) or absent in some inner hair cells in the heterozygous KI cochlea (Fig. 5b, b’). Hair cells co-expressing *Atoh1* and *Neurog1* displayed heterogeneity in the extent of *Fgf8* expression. In the homozygous KI mice, *Fgf8* was not expressed in the cochlea like *Atoh1* (Fig. 5b”, b”’, a”, a”’). In contrast, a few *Fgf8* positive inner hair cell precursors were identified in the *Atoh1* CKO cochlea in absence of *Atoh1* (Fig. 5a””, b””), indicating that *Neurog1* expression negatively affects *Fgf8* expression in both heterozygous and homozygous KI mice (Fig. 5b’,b”,b”’).

In contrast to *Fgf8*, other genes showed upregulation in heterozygous KI mice (Fig. 5c’,d’,e’,f’,g’,h’,i’,j’). *Neurod1* showed a more profound, albeit patchy expression in the homozygous KI cochlea comparable to *Neurog1* expression (Fig. 5c”, c”’,d”, d”’). In contrast, *Neurod1* was undetectable by *in situ* hybridization in the *Atoh1* CKO cochlea (Fig. 5d””) indicating that either *Atoh1* or *Neurog1* is needed to drive the expression of *Neurod1* in hair cells. Another bHLH gene, *Nhlh1*, which has been claimed to be regulated by both *Neurod1* and *Atoh1* [35], [39], was expressed in patches of organ of Corti cells in the apical half of the cochlea but was absent in the base of homozygous KI mice (Fig. 5e”,e’”). *Nhlh1* was absent in the *Atoh1* CKO mice (Fig. 5e””). Clearly, the expression of *Neurod1* and *Nhlh1* in the organ of Corti cells of the homozygous KI cochlea was driven by misexpression of *Neurog1* under the endogenous *Atoh1* promoter. These data showed that expression of *Neurog1* affected normal gene expression in differentiating hair cells or in undifferentiated hair cell precursors and could thus progressively alter the organ of Corti differentiation.

Since *Neurog1* misexpression in hair cell precursors lacking Atoh1 can elicit expression of some genes within organ of Corti precursors (Fig. 5d”, d””, e”, e””), we next wanted to evaluate a more extensive panel of genes that affect the prosensory development of the inner ear. We investigated *Jag1*, a Notch ligand, which is required for prosensory formation and the function of Notch [40] downstream of *Rbp-j* [41], [42]. *Jag1* displayed a patchy expression pattern with a gradient from apex to base forming a ‘rosette-like’ structure in the homozygous KI organ of Corti (Fig. 5f”, f”’, insert in f” and f”’). The presence of *Jag1* in a cluster of cells may indicate the potential for formation of mosaic cellular pattern of the organ of Corti. However, unlike the other Notch ligands, early *Jag1* expression specifies the prosensory domain with a subsequent expression in the adjacent supporting cells [40], [43], [44]. In contrast, *Jag1* expression in the *Atoh1* CKO mice was nearly absent (Fig. 5f””), except for a limited expression in the apical tip (data not shown). *Hes5*, a downstream mediator of Notch [45], [46], was also differentially expressed in the heterozygous and homozygous KI mice (Fig. 5g–g”’). Homozygous KI mice showed expression of *Hes5* only in the apex of the cochlea (Fig. 5g”, g”’). Absence of *Jag1* and *Hes5* in the *Atoh1* CKO (Fig. 5f””, g””) indicated that, removal of *Atoh1* limited prosensory epithelium formation and ultimately resulted in the formation of ‘flat epithelium’. In the homozygous KI mice *Neurog1* initiated some degree of prosensory development resulting in survival of some organ of Corti cells, including a limited, patchy expression of *Jag1* and *Hes5*.

We next examined the expression of *Gata3*, a zinc finger transcription factor expressed in the neurosensory precursors of the otic epithelium [47]. Some disparity in the *Gata3* expression pattern was observed between homozygous KI and *Atoh1* CKO mice (Fig. 5h–h””) such as patchy expression of *Gata3* next to organ of Corti precursors in the homozygous KI cochleae (Fig. 5h”). In addition, *Gata3* was moderately increased in the basal half of the cochlea in homozygous KI mice (Fig. 5h”’) in contrast to *Atoh1* CKO mice (Fig. 5h””). This patchy *Gata3* expression indicated some intercellular signaling between the organ of Corti-like clusters of cells that expressed *Neurog1*, *Neurod1*, *Nhlh1* and *Jag1* to surrounding cells. Therefore, *Neurog1* misexpression had a broader impact than just alterations of fate in the hair cell precursor and extended to the adjacent supporting cells.

We next wanted to understand if the effects of *Neurog1* misexpression impact other genes that affect the development of the organ of Corti. *Fgf10* is expressed in the GER, medial to the developing organ of Corti [48]; whereas, *Bmp4* defines the lateral boundary of the organ of Corti destined to become the Hensen’s and Claudius cells [49], [50]. It was reported previously that the expression of *Fgf10* and *Bmp4* changed and downregulated in the absence of a differentiated organ of Corti, approximated toward each other as the organ of Corti cells degenerate [22]. We therefore wanted to investigate the expression pattern of these two organ of Corti flanking molecular markers in the presence of *Neurog1* positive organ of Corti-like cells in the homozygous KI mice (Fig.5i–i”’, j–j”’). We found that the differentiated organ of Corti in wild-type and in heterozygous KI cochlea were flanked by *Fgf10* medially (neural side) and *Bmp4* laterally (abneural side) (Fig. 5i, i’, j, j’). In the homozygous KI cochlea, *in situ* data demonstrated lateral expansion of *Fgf10* and medial expansion of *Bmp4*, extending into the areas of the organ of Corti between the patches of organ of Corti cells (Fig. 5i”, i”’, j”, j”’). These data suggested that presence of *Neurog1* positive organ of Corti cells in homozygous KI mice affected the expression patterns of these molecular markers in more distant cells belonging to the GER and the Claudius cells, indicating the existence of a thus far uncharacterized feedback loop emanating from the organ of Corti. This pattern of *Bmp4* expression correlated with medial expansion of Claudius-like cells between remaining *Neurog1* positive organ of Corti cells observed in the SEM (Fig. 3c,d,e). As previously reported [22], in *Atoh1* CKO mice, *Bmp4* and *Fgf10* expression was also decreased in a base to apex gradient but no medial and lateral expansions into the shrinking organ of Corti were observed (Fig. 5i””, j””).

We also investigated expression of *miR-96* which is essential for inner ear neurosensory development [51]. *MiR-96, a miR-183* family member, is expressed in inner ear neurons at an early stage and later in sensory epithelia [34]. Mutation of *miR-96* results in nonsyndromic progressive hearing loss in humans and mice [51]. We observed retention of *miR-96* expression in the organ of Corti precursor cells in the apex of homozygous KI mice in contrast to diminished expression in *Atoh1* CKO mice (Fig. S4c,c’,d,d’). We then examined *miR-124* expression, which is normally associated with neuronal differentiation [52], to determine whether it is misexpressed in hair cells in KI mice. *In situ* hybridization of *miR-124* showed many positive neurons were present in homozygous KI mice, particularly in the basal half compared to *Atoh1* CKO spiral ganglia (Fig. S4g and h). However, *miR-124* expression was not found in hair cells of heterozygous or homozygous KI mice.

In conclusion, while *Gata3*, *Jag1*, *Fgf10* and *Bmp4* were expressed prior to and independent of *Atoh1* in and around the developing organ of Corti, misexpression of *Neurog1* in cochlear hair cells generated a considerable amount of expression changes within and outside the organ of Corti. This is vastly different from changes in the cochlea associated with the simple loss of *Atoh1* and indicates the impact of signaling by the *Neurog1* positive cells to the surrounding cells in the cochlea.

### Replacement of *Atoh1* with *Neurog1* led to patchy degeneration of the organ of Corti in homozygous KI mice

**Figure 6:**
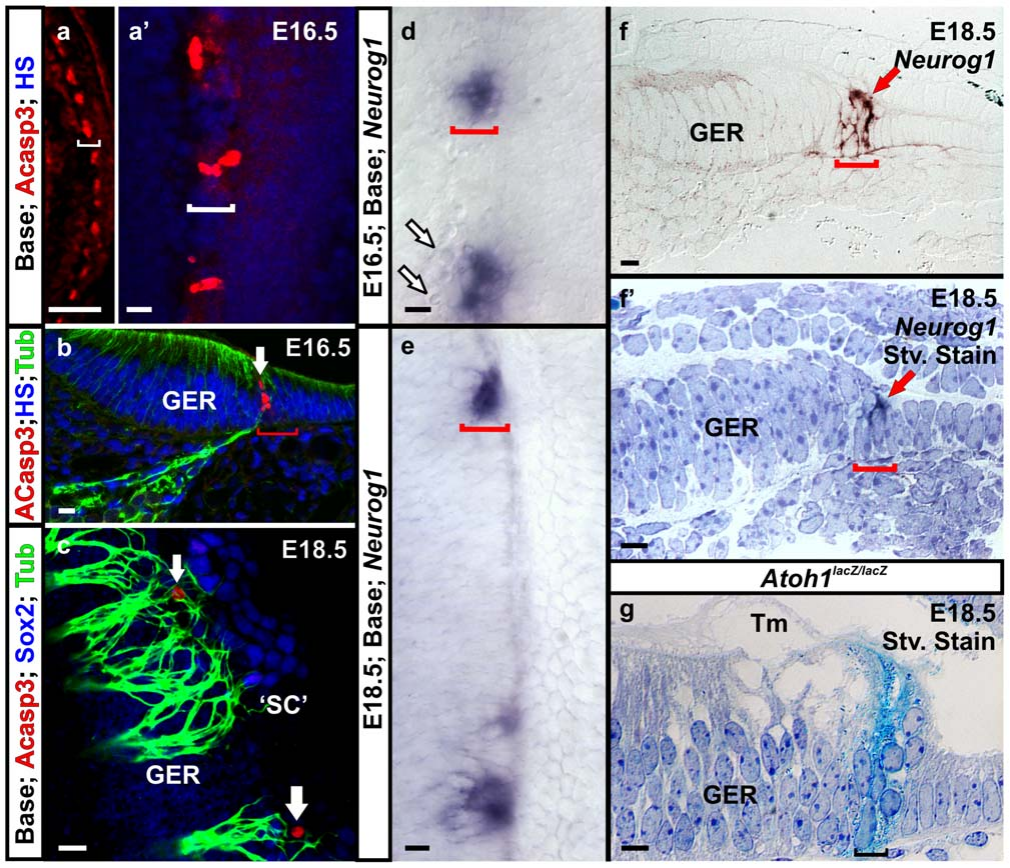
Replacement of *Atoh1* with *Neurog1* results in patchy degeneration in the organ of Corti cells. Immunochemistry of activated Caspase3 in homozygous KI mice reveal the labeling of patches of cells in the putative organ of Corti, mostly in the basal half of the cochlea at E16.5 (a, a’,b). These dying cells are typically 1-2 cells in one patch situated more medially (neural side) of the organ of Corti (white arrow in b, c, d), typically on the medial edge of the Sox2 positive presumed supporting cells (c), most likely the precursors of inner hair cells or the first row of outer hair cells (a-c). *Neurog1 in situ* positive patches of cells in the homozygous KI mice are progressively reduced from E16.5 to E18.5 particularly in the base (d and e) consistent with the appearance of activated Caspase3 labeled cells in the base. *Neurog1* positive cells shown in thin plastic sections performed after whole mount *Neurog1 in situ* hybridization (red arrows in f and f’) form compact clusters of 2-3 cells. The histological localization of the *Neurog1* positive cells in homozygous KI mice is comparable to LacZ positive cells in the *Atoh1* null mice (f’, g). ACasp3, activated Caspase3; GER, greater epithelial ridge; HS, Hoechst stain; Stv. Stain, Stevenel’s blue staining; ‘SC’, putative supporting cells expressing Sox2; Tm, tectorial membrane. ‘[‘ indicates position of the putative organ of Corti. Bar indicates 10 µm except 100 µm in a.

**Figure 7:**
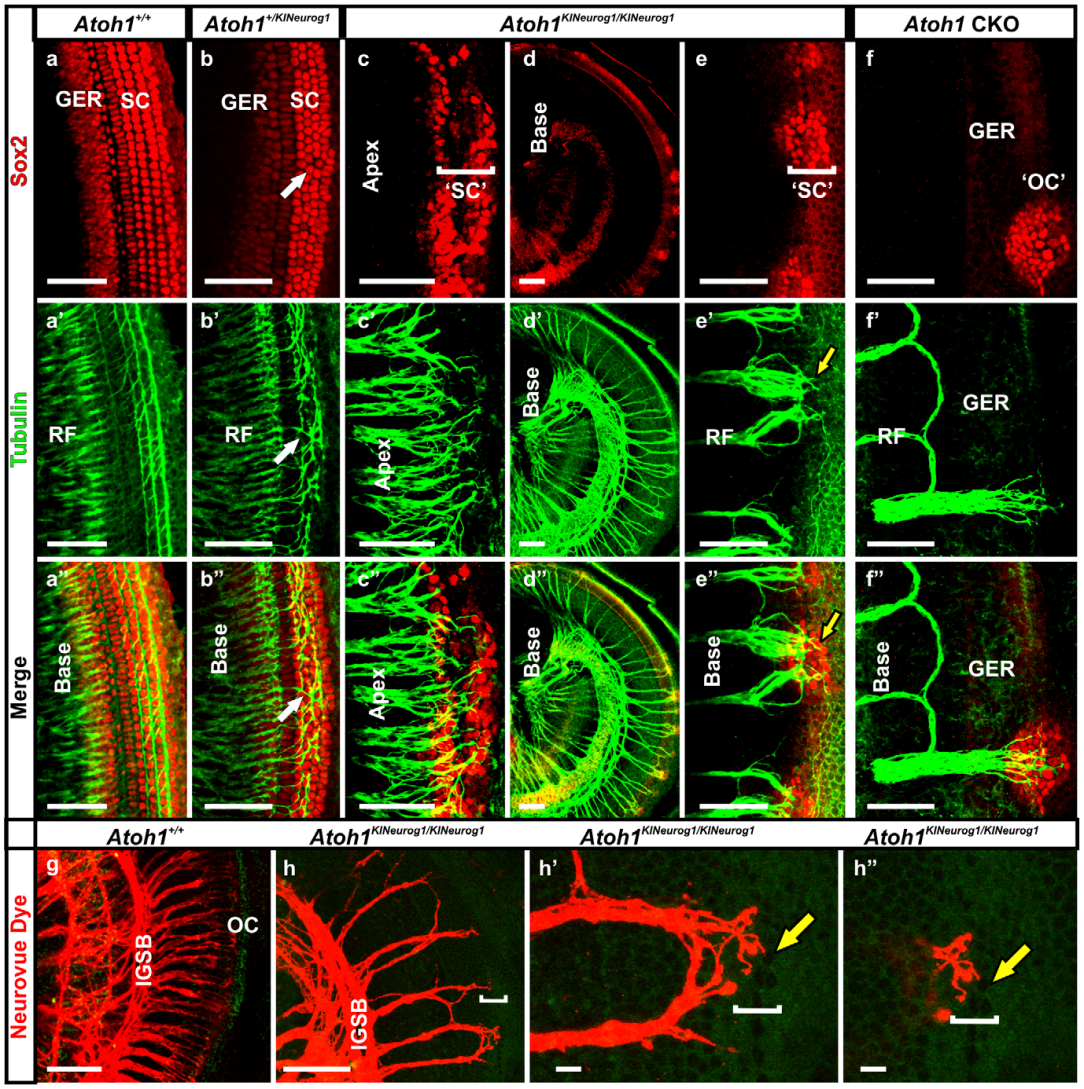
*Neurog1* expression in organ of Corti cells affects afferent and efferent innervation. In E18.5 wild-type mice, Sox2 is present in the GER and in all supporting cells (a) and many radial fibers project to inner hair cells. Anti-Tubulin antibody labeling shows Type II fibers projecting in a regular pattern to outer hair cells (a’) between the Sox2 immunopositive supporting cells (a”). Heterozygous KI mice reveal some disorientation in Sox2 positive supporting cells with disorganized projection of type II fibers between the disorganized supporting cells (arrows in b, b’, b”). In homozygous KI littermates Sox2 positive cells are organized as multiple patches along the organ of Corti with a gradient of decreased density from apex to base (c-e”). Tubulin immunolabeling reveals fibers reaching to these patches and extending between Sox2 positive cells (arrows in e’ and e”). *Atoh1* CKO mice have few occasional patches of Sox2 positive cells (f) and tubulin positive fibers mostly form loops with some reaching with rare projections to Sox2 positive cells of the organ of Corti (compare e’ and f’). Neurovue dye tracing of efferent innervation from the brainstem shows formation of intraganglionic spiral bundles (IGSB) in homozygous KI mice comparable to wild-type littermate (compare g and h). Multiple efferents in the homozygous KI mice enter into the undifferentiated organ of Corti (h-h”) where fibers ramify apparently between the remaining patches of organ of Corti cells (arrows in h’,h”). GER, greater epithelial ridge; OC, organ of Corti; ‘OC’, presumed organ of Corti in *Atoh1* CKO; RF, radial fibers; SC, supporting cells; ‘SC’, putative supporting cells expressing Sox2; ‘[‘ indicates position of the putative organ of Corti. Bar indicates 50 µm in all except 100 µm in d-d”, g, h and 10 µm in h’ and h” .

Since the gene expression and SEM data revealed patches of microvilli bearing organ of Corti cells, we next wanted to investigate the fate of the cells between these patches. Previous work had shown that most organ of Corti cells die via apoptosis in *Atoh1* CKO or in *Atoh1* null mice [22], [53]. Immunochemistry revealed the presence of activated-Caspase3 in patches of organ of Corti cells mostly in the basal half of the cochlea around E16.5 (Fig. 6a, a’, b). The degeneration in the base of the cochlea correlated with the finding of fewer *Neurog1* positive patches of organ of Corti cells in the base of homozygous KI mice (Fig. 6d). We also found limited activated-Caspase3 positive cells at E18.5 in homozygous KI mice which were localized medial to Sox2 immunopositive presumed supporting cells (Fig. 6c). At E18.5 Sox2 positive cells were present both in the GER and in the supporting cells in control mice (Fig. 7a). These activated-Caspase3 positive cells were also innervated by the radial fibers shown with Tubulin immunochemistry (Fig. 6b, c). *Neurog1* positive organ of Corti-like cells in the homozygous KI cochlea gradually declined from E16.5 to E18.5 (Fig. 6d,e) as probably the cells negative for *Neurog1* died, similar to the fate of most organ of Corti cells in *Atoh1* null mice [53], [54]. Due to postnatal lethality of the mice we could not determine the fate of the remaining *Neurog1* positive cells. We suspect that continued reduction of *Neurog1* expressing organ of Corti cells will happen in neonates comparable to *Atoh1* null mice, which result in the eventual death of all Atoh1-negative hair cell precursors. Interestingly, this process of organ of Corti cell death was apparently delayed compared to simple *Atoh1* null mice where this was nearly completed at birth [53], [54], suggesting a possibly transient but limited rescue of organ of Corti cells by *Neurog1*.

As whole mount data suggested that particularly the more medial (neural side), likely representing inner hair cell progenitors, die first in a given patch (Fig. 6b,c, arrows in b, c, d), we sectioned a *Neurog1 in situ* reacted cochlea to better understand the distribution of *Neurog1* positive cells in the homozygous KI mice. As with *Atoh1* null mutants that express lacZ across a 1-2 cell wide band from the basilar membrane to the scala media lumen (Fig. 6g), we found somewhat similar looking cells that were *Neurog1* positive without being able to determine whether these cells were inner or outer hair cell precursors (Fig. 6f, f’).

### Misexpression of *Neurog1* caused survival of afferent and efferent innervation in homozygous KI mice supported by Ntf3 expression

Absence of differentiating hair cells resulted in extensive neuronal death in *Atoh1* CKO mice [22] probably due to the absence of neurotrophins needed to support the neurons in the ‘flat epithelium’ [22]. Moreover, the remaining neurons mostly never reach into the ‘flat epithelium’ of *Atoh1* null [22] or chemically ablated mice [55]. We wanted to investigate whether organ of Corti cells that misexpressed *Neurog1* behaved differently from those cells of the ‘flat epithelium’. Tubulin immunochemistry demonstrated the distribution of radial fibers projecting to the patchy organ of Corti cells with the progressively increasing gradient from base to apex being associated with patches of the homozygous KI mice (Fig. 7c–c”,d-d”,e-e”). In contrast to the *Atoh1* CKO mice, where the majority of the fibers did not reach the presumed organ of Corti (Fig. 7f’; [22]), homozygous KI mice showed a substantial increase in radial fiber density in similar regions of the cochlea with their terminals reaching deep into the organ of Corti-like cells (Fig. 7c’, d’, e’).

*Sox2* is an early marker of the prosensory domain of the developing cochlea and required for organ of Corti development [56]. Sox2 immunochemistry showed distribution of Sox2 positive cells in the GER and in the supporting cells in E18.5 wild-type mice (Fig. 7a). We found clusters of Sox2 immunopositive cells in the cochlea of E18.5 homozygous KI mice with a gradient from apex to base (Fig. 7c–d) which contrasted to the occasional Sox2 positive cells found in the apex of *Atoh1* null mutants [57] or in *Atoh1* CKO mice (Fig. 7f; [22]). Radial fiber terminals projected to the Sox2 positive areas in the homozygous KI cochlea; whereas, in *Atoh1* CKO mice these fibers formed loops before reaching the proximity to GER with very occasional fibers reaching the few Sox2 positive cells of the organ of Corti (Fig. 7c–c”,d–d”,e–e”; [22]). The radial width of the Sox2 positive patches in the GER was reduced in the homozygous KI mice compared to wild-type littermate (Fig. 7a, c–e), which corresponded to the loss of other markers of the GER such as *Fgf10* (Fig. 5h”, h”’, S4). The possible interactions between Sox2, Fgf10 and Bmp4 remain to be explored.

Interestingly, Sox2 immunofluorescence in the heterozygous KI mice revealed disorganization in the supporting cell layer morphology along with reduced intensity of Sox2 in the GER (Fig. 7b). Tubulin immunostaining also showed the disorganization of the type II fibers with aberrant projections of radial fibers both toward the apex and base of the cochlea (Fig. 7b’). This aberrant fiber projection was related to the abnormalities found in the outer hair cells and supporting cells in the heterozygous KI mice (Fig. 4). This phenotype resembled somewhat the defects reported in *Prox1* null mice [58] and could indicate interaction of ingrowing fibers with supporting cells. However, *Prox1* expression in the E18.5 heterozygous KI mice showed a near normal pattern by *in situ* hybridization (Fig. S5b’) but displayed aberrant expression in a later stage (Fig. 4a’, a”). In contrast, the homozygous KI mice showed diminished levels of both *Prox1* mRNA and protein where the signal was limited to only a few patches in the cochlea (Fig. S5c, c’, d). Furthermore, *Prox1* was clearly expressed in the basal turn of homozygous KI cochlea compared to complete absence in the *Atoh1* null cochlea [57].

Previous work has established that efferent fiber growth is more truncated than afferent fiber growth in *Atoh1* null mice [22] suggesting that neurons with limited support from a non-existing organ of Corti cannot sustain proper fiber extension [59]. Efferent innervation was examined using Neurovue dye injections into the brainstem in the homozygous KI mice. We found profound outgrowth of efferent fibers, including well-formed intra-ganglionic spiral bundles (Fig. 7h). Surprisingly, the efferent fibers in the homozygous KI mice projected into the remaining organ of Corti patches where they ramified around unidentified organ of Corti cells (Fig. 7h’, h”). In summary, both afferent and efferent fibers were retained in patches in the homozygous KI mice and extended processes into *Neurog1* positive clusters of organ of Corti cells which were likely in the center of the Sox2 positive patches.

**Figure 8:**
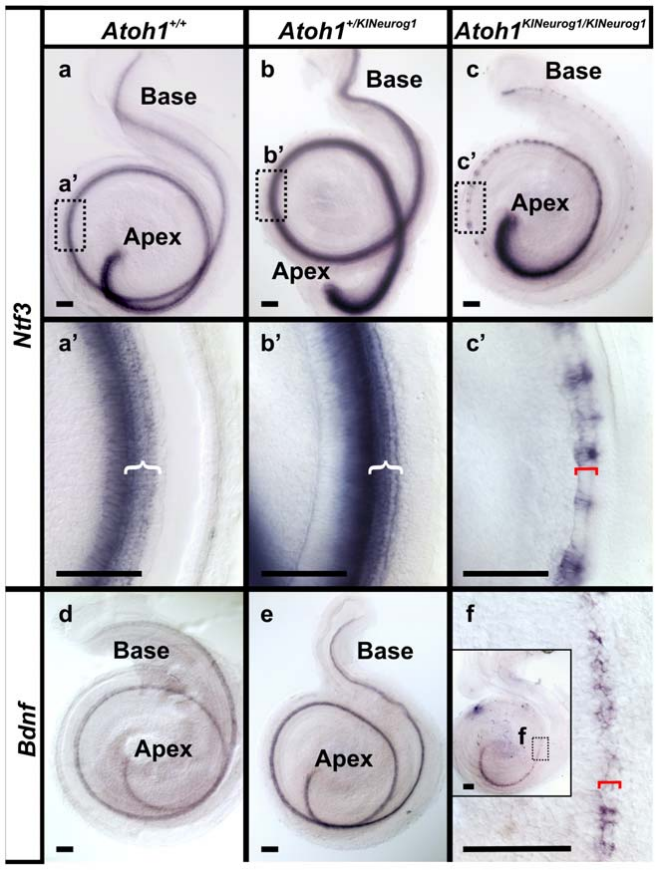
Residual *Ntf3* expression in the patchy organ of Corti cells may support restricted innervation. *Ntf3* expression is uniform throughout the organ of Corti but appears more prominent in heterozygous KI mice compared to the wild-type littermate (a, a’, b, b’). In contrast, homozygous KI mice exhibit patchy *Ntf3* expression in the middle and base, but continuous in the apex and in basal tip of the cochlea (c,c’), consistent with Sox2 distribution and the pattern of residual innervation. In contrast, *Bdnf* which is predominantly expressed in the hair cells is somewhat elevated in heterozygotic KI mice compared to wild-type littermates (d,e) but shows expression only in the apical turn in homozygotic KI mice (f; insert in f). ‘{‘ indicates the area of differentiated organ of Corti ‘[‘ indicates the putative organ of Corti in the homozygous KI mice. Bar indicates 100 µm.

Two types of neurotrophins, *Ntf3* and *Bdnf,* provide trophic support to developing inner ear sensory neurons [60]. *Bdnf* is responsible for the innervation of embryonic vestibular epithelia whereas *Ntf3* predominantly supports the basal turn spiral neurons. We therefore investigated the expression of *Ntf3* and *Bdnf* in both heterozygous and homozygous KI mice by *in situ* hybridization (Fig. 8, S6). A profound expression of *Ntf3* was found in heterozygous KI cochlea at E18.5 compared to wild-type littermates, as well as *Ntf3* being localized to the clusters of organ of Corti precursors in homozygous KI cochlea (Fig. 8a–c’). These results contrasted with the previous report of lack of *Ntf3* expression in the absence of hair cell differentiation in *Atoh1* CKO [22]. Misexpression of *Neurog1* enhanced the *Ntf3* expression in the organ of Corti precursors. The presence of *Ntf3* apparently provided support to radial fiber growth in those patches of cells in the homozygous KI mice (Fig. 7c’,d’,e’, 8c,c’) consistent with the functional role of *Ntf3* in spiral ganglion cell development and maintenance [60], [61], [62]. In contrast, the innervation was greatly reduced in the *Atoh1* CKO, a fact that corresponded well to reduced expression of *Ntf3* (Fig. 7f’, [22]).

Unlike *Ntf3*, *Bdnf* expression remained unchanged in E18.5 homozygous KI mice (Fig. 8f) as *Bdnf* expression is possible even in the absence of *Atoh1* mediated differentiation of hair cells [36] and is likely due to its complex promoter system that does not appear to be exclusively dependent on *Atoh1* expression [63], [64]. *Bdnf* expression persisted only in the apex of the homozygous KI cochlea which was consistent with the dense apical innervation reported in in the *Atoh1* null [36] and in *Atoh1* CKO mutants [22]. Analysis of earlier stages (E13.5) revealed *Bdnf* expression in the delaminating neurons as well as in the hair cells of the vestibular epithelia, being most profound in the canal crista and in the apex of the cochlea in wild-type mice (Fig. S6d, d’). Likewise, both the homozygous KI and *Atoh1* CKO mice showed expression of *Bdnf* in the canal cristae and in the apex of the cochlea (Fig. S6e–f’). *Bdnf* was absent in the utricular and saccular hair cells, whereas it was profoundly expressed in the delaminating neurons near the utricle and saccule in both homozygous KI and *Atoh1* CKO mice (Fig. S6e–f’). This is consistent with previous work which showed that in *Neurog1* null mice no neurons delaminated and *Bdnf*-lacZ positive cells were restricted to the sensory epithelia of utricle and saccule [25]. Homozygous KI mice showed no apparent alteration in expression of *Bdnf* relative to *Atoh1* CKO (Fig. S6e–f’, 7f; [22]) or *Atoh1* null mice [36]. Therefore, we demonstrated that expression of *Ntf3* particularly at E18.5 in the clusters of organ of Corti-like cells in homozygous KI mice (Fig. 8c,c’; S6h,h’), provided the molecular basis for the enhanced afferent and efferent innervation of these patches. Future work in viable adult mice with at least one allele carrying the *Neurog1* KI combined with a floxed *Atoh1* allele is needed to investigate the long term fate of cochlear hair cell innervation.

**Figure 9:**
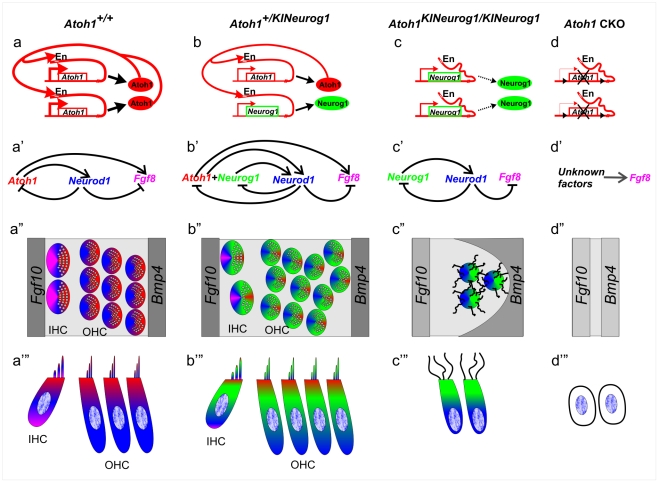
*Atoh1* promoter driven *Neurog1* differentially regulates downstream genes and affects organ of Corti cell differentiation. This diagram shows the interactions of Atoh1 protein with the its enhancer to increase levels of *Atoh1* mRNA expression (a). Atoh1 in part regulates *Neurod1* and presumably also *Fgf8* (a’, [74]) whereas *Neurod1* in turn suppresses *Atoh1* and *Fgf8* as reported previously in *Neurod1* null mice [24] and shown here. In wild-type mice inner hair cells (IHC) express all three genes (color coded as, red, *Atoh1*; blue, *Neurod1* and lilac, *Fgf8*; a’) and outer hair cells (OHC) express *Atoh1* and *Neurod1* but not *Fgf8* (red and blue; a’, a”). In heterozygous KI mice (*Atoh1^+/KINeurog1^*) (b-b”’) enhancer activation of *Atoh1* as well as activation of the *Neurod1* and *Fgf8* will be retained with expression of *Atoh1*. However, Neurog1 protein mediated enhanced expression of *Neurod1* suppresses *Atoh1* and *Fgf8* more strongly. Consistent with the proposed regulation changes are our observed changes in mRNA expression pattern and the effects on hair cell development in heterozygous KI mice such as extra rows of outer hair cells, misalignment and progressive loss of inner hair cells (b”,b’”). In heterozygous KI mice, all four genes including *Neurog1* are expressed in IHC (green) but have reduced expression of *Atoh1* and *Fgf8* (b”, b’”) and the three genes are expressed in OHC (b”, b’”). In homozygous KI mice (*Atoh1^KINeurog1/KINeurog1^*) there is no *Atoh1* expression and thus no activation of the *Atoh1* enhancer (c). Therefore, enhanced expression of *Atoh1* driven by the Atoh1 protein binding to the enhancer will be disrupted, leaving only non-Atoh1 protein mediated activation to drive *Neurog1* expression. Neurog1 protein mediated *Neurod1* expression apparently suppresses all *Fgf8* expression (c’). Homozygous KI mice show undifferentiated organ of Corti cells (which express only *Neurog1* and *Neurod1*) that may survive but form only microvilli (c”, c’”) and are surrounded by a reduced expression of *Bmp4* (c”). In the *Atoh1* CKO mice (d) the recombined Atoh1 locus (At) does not produce a protein able to activate the enhancer or expression of *Neurod1.* However, a limited expression of Fgf8 is possible indicating that other factors are involved in regulating that gene. The reduced expression domains of *Fgf10* and *Bmp4* approximate each other (d”) and no organ of Corti cell remains in the ‘flat epithelium’ (d”, d’”).

In summary (Fig. 9), our data suggested that we obtained expression of *Neurog1* under the control of the *Atoh1* promoter as predicted. Our expression, morphological and histological data suggested that co-expression of *Neurog1* with *Atoh1* in the heterozygous KI mice resulted in mild aberrations of hair cell and supporting cell development, indicating that *Neurog1* altered the hair cell phenotype beyond a haploinsufficiency of *Atoh1*. Homozygous KI mice showed rescue of some undifferentiated organ of Corti cells at least until P0. These cells had a distinct gene expression profile, showed an unusual morphology, attracted more afferent and efferent neurites than simple *Atoh1* null mice, but did not differentiate either as neurons or as hair cells in the organ of Corti.

## Discussion

Past work has shown that replacement of certain bHLH genes with other paralogs can reveal functional equivalence [1], [65], [66]. This was largely attributed to redundancy of signaling possibly due to the similarity of E-box sequences recognized by the different bHLH proteins [14], [67]. In essence, it was proposed that limited redundancy of expression in the PNS requires a key-lock action of a specific bHLH gene whereas in the CNS the general bHLH gene action combined with channeling effects of the cellular context suffice. Among bHLH genes, *Atoh1* is unique in that it is expressed in both proliferating precursors and/or postmitotic differentiating cells [16], [25], [65], [68]. In addition, *Atoh1*/*atonal* is the only bHLH gene known to be functionally conserved across phyla: the fly *atonal* gene functions in mouse and the mouse *Atoh1* gene functions in flies [69]. Hair cells belong to an ancient group of neurosensory cells that have split in vertebrate ancestors into two cells, a neuron and an axonless hair cell [70]. There is a functional bifurcation with the derived bHLH gene, *Neurog1,* driving neuronal development [18], [23] whereas the conserved bHLH gene, *Atoh1*, driving hair cell development [14]. Generating a mouse in which *Neurog1* and *Atoh1* can be co-expressed in the same cell through their regulation by the endogenous *Atoh1* promoter (*Atoh1* ^+/*KINeurog1*^ *)* could result in three possible phenotypes:

1. Hair cells could be primed to respond exclusively to *Atoh1* and will develop normally, like in a simple *Atoh1^+/-^* mouse [17].
2. All or a subset of hair cells (in particular those who have a lineage relationship with neurons [24], [25]) could respond to *Neurog1* to differentiate as neurons.
3. All or a subset of hair cells (in particular those who have a lineal relationship with neurons [24], [25]) could differentiate as a hybrid between neurons and hair cells, forming essentially primary sensory cells with their own axon, much like sensory cells in many invertebrates, including the *atonal* dependent fly mechanosensory cells [71].

Beyond these possible outcomes, we wanted to understand further the necessity of availability of Atoh1 protein to drive hair cell differentiation without activation by a positive feed-forward mechanism that utilizes a *cis*-enhancer element. We choose to eliminate Atoh1 protein (Fig. 9) through its substitution by another bHLH gene presumably unable to bind to the *Atoh1*-specific enhancer E-box [3]. In our KI mouse the *Atoh1* coding sequence is replaced by *Neurog1*, a closely related bHLH gene [14]. We analyzed the effects of *Neurog1* misexpression in the homozygous and heterozygous KI mice on the development of the ear. Our data show that not only hair cell development is impaired by substitution of *Atoh1* with its *Neurog1* paralog but *Neurog1* does modify the hair cell phenotype even when *Atoh1* is present. The impact of *Neurog1* misexpression is not just localized to the hair cells, but there is also a wider disturbance of both the morphology and expression pattern in the adjacent supporting cells as well as the more distal cells in the organ of Corti, GER and spiral ganglion.

### Neurog1 protein can transiently support the viability of some organ of Corti and surrounding cells, but cannot initiate their full differentiation

In homozygous KI mice, patchy *Neurog1* positive cells remain in the base and middle turn of the organ of Corti, distinct from the *Atoh1*-lacZ distribution in *Atoh1*-null cochlea where all organ of Corti cells seem to die over time [22], [53]. Previous work had identified cross-regulation between *Atoh1* and *Neurog1* in the spinal cord [2] and the ear [22], [24]. Specifically, in *Atoh1* null mice there is an expansion of *Neurog1* [24] and *Neurod1* [22] in the utricle and saccule. We also found a more profound expression of *Neurog1* in the delaminating neuroblasts in the utricle and saccule in the E14.5 homozygous KI mice as well as in the vestibular sensory epithelia and in the mid-base region of the cochlea. As expected, absence of a putative negative feedback loop in neurons, due to the absence of Atoh1 protein, resulted in enhanced expression of *Neurog1* in the delaminating neuroblasts or common neurosensory precursors. However, the reduced differentiation of the organ of Corti appears to be unable to sustain neuron retention.

SEM investigations reveal the development of tall microvilli among some of these clusters of organ of Corti cells in homozygous KI mice, a novel feature in the absence of hair cell differentiating factor Atoh1. The appearance of these cells in more lateral areas of the organ of Cort is suggestive of outer hair cells precursors. This assumption is consistent with the more medial loss of cells in the organ of Corti observed with activated caspase3. *Neurog1* misexpression supports these patches of cells to survive as a modified precursor cell with some rudimentary stereocilia. Whether these surviving cells relate to a specific hair cell precursor type has not yet been established.

The *Neurog1* positive patches of organ of Corti precursor cells can drive expression of downstream genes like *Neurod1* and *Nhlh1* [18]. Comparable to other mutants like *Pou4f3*, where some pillar cells form due to interaction with short-lived hair cells [72], replacement of *Atoh1* with *Neurog1* in homozygous KI mice can apparently modulate interactions with surrounding cells, possibly through expression of delta/notch ligands and effector genes such as *Jag1 and Hes5,* resulting in enhanced viability of the additional non-*Neurog1* positive cells in these organ of Corti patches. This extent of differentiation has not been reported in *Atoh1* null [17] or *Atoh1* CKO [22] mice where *Jag1* shows faint expression only in the apex of the cochlea. In addition to *Jag1*, the expression of *Sox2 and Prox1,* presumably in supporting cell precursors in the center of a rosette-like cluster of organ of Corti cells, indicates intercellular interactions to specify to some extent organ of Corti cell differentiation. In contrast to the basal expression in the homozygous KI mice, Sox2 and Prox1 are only found in the apex of *Atoh1* null mice [22], [57] with very few occasional patches of Sox2 in the middle turn.

*Neurog1* misexpression also alters expression of *Fgf10* and *Bmp4* which are defining the medial and lateral boundaries of the cochlea, respectively [48], [49]. BMP signaling is necessary for prosensory specification as shown in double-mutant mice of BMP receptors, *Alk3/Alk6* [50]. A previous report in *Atoh1* CKO mice showed that subsequent to organ of Corti degeneration, *Fgf10* and *Bmp4* expression diminished and more closely spaced to each other [22] by reducing the area of the putative organ of Corti as revealed by *Gata3* expression. In homozygous KI mice survival of organ of Corti-like patches of cells modify the expression of *Bmp4*, *Fgf10* and *Gata3*. *In situ* hybridization of these markers revealed undulation of the boundaries adjacent to the remaining organ of Corti patches. Combined with the SEM data, this suggests that *Bmp4* positive domains of non-sensory cells (mostly Claudius cells) expand medially between the remaining sensory patches, transforming the cells between patches into a ‘flat epithelium’ [73]. These data suggest a short range interaction of the organ of Corti cells with surrounding cells expressing *Bmp4* and *Fgf10* that has not been recognized thus far.

### Neurog1 protein interferes with Atoh1 signaling in developing hair cells of heterozygous KI mice

In heterozygous KI mice, *Neurog1* recapitulates the spatio-temporal expression of *Atoh1* resulting in co-expression of *Atoh1* and *Neurog1* in hair cells. We took advantage of the viability of these mice to analyze the extent and diversity of hair cell differentiation at post-natal stages. Immunochemistry of Myo7a, Prox1 and Tubulin revealed the progressive disorganization of both hair cells and supporting cells in heterozygous KI mice. SEM investigations correlated with the finding of immunochemistry. We found disoriented organ of Corti cell formation. Ooccasionally some inner hair cells were replaced by pillar cells with a gradual loss of some inner hair cells and pillar cells in later stage (P26). The phenotype in the heterozygous KI mice differs from haploinsufficient effects of *Atoh1^LacZ/+^* [17] or *Pax2-cre; Atoh1* ^f/+^ mice [22]. This suggests that Neurog1 protein alters Atoh1 signaling. This might occur prior to E-box binding, through reduced availability of E-proteins, at E-box binding by sterically hindering Atoh1-E-protein dimer binding, or by regulating different downstream genes that interfere with Atoh1 downstream signals. Our data provides evidence that co-expression of *Atoh1* and *Neurog1* prematurely upregulates expression of *Neurod1* and *Nhlh1* in hair cells but delays *Atoh1* and *Fgf8* expression in hair cells and alters *Neurog1* expression in delaminating sensory neurons. Clearly, co-expression of *Neurog1* with *Atoh1* can modulate *Neurog1* downstream gene expression, indicating that perhaps the latter aspect is the most prominent. Our data suggest a negative feedback loop of Neurog1 on hair cells, somewhat comparable to that observed in other systems [19] and possibly in part mediated by Neurod1 (Fig. 9). Consistent with this is the effect of KI insertion on *Fgf8* expression in inner hair cells. *Fgf8* becomes prematurely upregulated in the absence of *Neurod1* [19] but is downregulated in the heterozygous KI mice and eliminated in KI homozygous mice (Fig. 9). These data are in agreement with the presence of *Neurod1* specific E-box sequence in an *Fgf8* intronic enhancer region that requires further functional verification [74]. Our data suggest that *Neurog1* contributes to and alters the expression profile of inner ear hair cells resulting in a modified hair cell phenotype. How these expression changes relate causally to the later defects in hair cell ultrastructure and viability remains to be established.

### Patches of organ of Corti cells in homozygous KI mice maintain some innervation through enhanced neurotrophin expression

It has been long hypothesized that inner ear hair cells are the attractor for the growth of afferent innervation by secreting neurotrophins [62], [75]. However, other reports suggest that initial afferents can grow in the absence of differentiated hair cells and without the presence of hair cell- and supporting cell-derived neurotrophins [22]. Nevertheless most of the afferents and efferents are lost before birth in the absence of hair cells [22]. We show that if *Neurog1* is substituted into the *Atoh1* locus, organ of Corti-like patches of cells remain that do not fully differentiate as hair cells but are densely innervated by both afferents and efferents. *In situ* hybridization shows the presence of *Ntf3* expressing clusters of organ of Corti cells which may be the factor responsible for the remaining innervation, consistent with the more prominent role of *Ntf3* in early cochlear afferent support [60], [61]. However, *Bdnf* expression persists in the apex as reported previously in *Atoh1* null mice, and may provide the additional support of dense innervation that remains in the apex of *Atoh1* null mice [22] and in homozygous KI mice. Analysis of neuronal markers such as *Fgf10*, *Prox1*, *miR124* in the KI mice demonstrates survival of the spiral ganglia in middle turn of the cochlea which are almost completely lost in *Atoh1* null [36] and in *Atoh1* CKO mice [22].

### Expression of *Neurog1* under *Atoh1* promoter control cannot change hair cell precursors to neurons

The limited ability of the *Neurog1* expression to transiently rescue patches of organ of Corti-like cells that show altered gene expression patterns in these and surrounding cells is a clear indication that the KI allele results in a protein that has some transcription factor signaling ability. However, this knockin of *Neurog1* into the *Atoh1* locus cannot initiate hair cell differentiation, but can modulate the organ of Corti precursors to survive as clusters of undifferentiated cells instead of leading to rapid and complete apoptosis as in *Atoh1* null [36] and in *Atoh1* CKO mice [22]. Despite this retention of immature patches of the organ of Corti, it is obvious that *Neurog1* cannot change the fate of organ of Corti precursors to differentiate as neurons. It remains to be seen if the Neurog1 effects are due to limited signaling ability of *Neurog1*, limited accessibility of Neurog1 specific E-boxes, the channeling of development through factors that control the expression of *Atoh1* (and thus *Neurog1* in the KI allele) or partial overlapping in *Atoh1*-associated downstream gene regulation.

In summary, our data show that *Neurog1* expression under endogenous *Atoh1* promoter control interferes with normal *Atoh1* signaling in the hair cells of heterozygous KI mice (Fig. 9). This is likely due to the negative feedback provided by the early and profound regulation of *Neurod1* in the heterozygous KI mice and a concomitant suppression of *Fgf8* and *Atoh1* expression, leading to disorganization and loss of hair cells. In homozygous KI mice the absence of Atoh1 protein likely limits the level of expression of Neurog1 which nevertheless can drive the expression of several downstream genes that result in partially viable organ of Corti cells with high density of innervation. These cells signal on their immediate and distant neighbors, changing expression profiles of several genes in patches of organ of Corti cells. What initiates cell death in between the patches of surviving organ of Corti cells requires further molecular characterization. Understanding why some organ of Corti precursors remain at least until birth while others die rapidly suggests a surprising level of genetic heterogeneity inherent within the hair cell population that could, if understood, help to minimize hair cell damage and thus delay hearing loss. Mice that combine a conditional knockout of the *Atoh1* allele with the *Neurog1* KI allele are currently being bred to verify the long term effects of the *Neurog1* expression on hair cells of adult mice.

## Supporting Information

Figure S1 ***Atoh1^KINeurog1^*** **construct.** This illustration shows the knockin construct where myc epitope-tagged *Neurog1* coding sequence was introduced in place of *Atoh1* coding region. An IRES-DsRed2 fragment was inserted 3′ to the coding region of *Neurog1* to show its expression with this reporter. The polyadenylation sequence (PAS) was preserved after the DsRed2. A second fragment that includes the pGK-neo selection cassette flanked by two loxP sites was inserted downstream to the *Neurog1* coding sequence (a, b). The restriction enzyme sites are shown by vertical lines. (E: EcoRI, H: HindIII) and the two probes used in Southern blotting are also indicated in b.
(TIF)

Figure S2 **Neurog1 and Myc-tag Protein expression.** Western blot analysis of Neurog1 and Myc-tag protein in the postnatal (P5 and P9) cerebella of heterozygous KI mice shows approximately correct molecular weight ∼25 and ∼36 kDa, respectively (a). However, wild-type littermates show similar sized bands, indicating some degree of non-specificity (b). β-Catenin antibody was used as the loading control in the western blot analysis (a,b).
(TIF)

Figure S3 ***Atoh1*** **expression is replaced by** ***Neurog1*** **in the cochlear nucleus and in cerebellum in** ***Neurog1*** **KI mice.** *In situ* hybridization shows downregulation of *Atoh1* expression in the cochlear nucleus and in cerebellum of the heterozygous KI mice (b, b’) and complete absence in the homozygous KI mice (c, c’) compared to wild-type littermate (a, a’). *Atoh1* is expressed in the proliferating precursors of the outer part of the external granule cell layer (insert in a’) which is maintained in some lobules in the heterozygous KI mice (b’). *Neurog1* is completely absent in the cochlear nucleus and very faintly expressed in the deep nuclei of the cerebellum of wild-type mice without any expression in the cerebellar cortex (d-d”). In contrast, both heterozygous and homozygous KI mice show *Neurog1* expression in the cochlear nucleus and in the proliferating external granule cell layer in cerebellum imitating the *Atoh1* expression (e-e”, f-f”). Replacement of *Atoh1* with *Neurog1* successfully recapitulates *Atoh1*- pattern both peripherally (ear) and centrally (cochlear nucleus and cerebellum). The smaller sized cerebellum in homozygous KI mice is demarked with black dotted line in c’ and d’. Red dotted lines in d”, e”, f” demonstrate the area of external granule cell layer (EGL). CB, cerebellum; CN, cochlear nucleus; CP, choroid plexus; IC, inferior colliculus. Bar indicates 500 µm.
(TIF)

Figure S4 ***Neurog1*** **KI mice show basal turn spiral ganglia and enhanced organ of Corti gene expression compared to** ***Atoh1*** **CKO mice.** *miR-96 in situ* hybridization shows expression in the hair cells in both wild-type and heterozygous KI mice at E18.5 (a,a’,b, b’). *miR-96* is expressed only in the apex of the homozygous KI cochlea whereas severely diminished in *Atoh1* CKO mice (c,c’,d,d’). *miR-124* is a neuronal marker expressed in all spiral ganglion cells (e-h). Homozygous KI mice retain neurons in the base that are almost absent in *Atoh1* CKO mice (g,h). *Neurog1* does not drive expression of *miR-124* in hair cells. Another marker, *Fgf10*, is uniform in wild-type heterozygous knockin mice (i,j) and shows presence of neurons in the base of homozygous knockin cochlea (k) compared to *Atoh1* CKO cochlea (l). *Fgf10* is also expressed in the GER in wild-type and heterozygous KI mice (i,j). Homozygous KI mice show reduction of *Fgf10* expression in the base, which is more profound in *Atoh1* CKO (k,l). Another marker, *Bmp4* is expressed in the Claudius cells defining the lateral (abneural) side of the developing organ of Corti. Simultaneous *in situ* hybridization of both *Fgf10* and *Bmp4* flank medial and lateral to the organ of Corti in wild-type and heterozygous KI cochleae (m,n). In homozygous KI mice, the patchy distribution of organ of Corti cells correlate with medial undulations of the *Bmp4* expression in the base of the cochlea. Both *Bmp4* and *Fgf10 in situ* signal are nearly absent in the *Atoh1* CKO base (o,p). Note that the spiral ganglia in the homozygous KI mice are removed to allow complete non-overlapping mounting of the cochlea (o). Spg, spiral ganglia. ‘OC’, putative organ of Corti in *Atoh1* CKO mice. ‘{‘ indicates the differentiated organ of Corti and ‘[‘ marks the presumed organ of corti in the homozygous KI mice. Green and yellow bar indicates *Fgf10* and *Bmp4* positive area, respectively. Bar indicates 100 µm.
(TIF)

Figure S5 ***Prox1*** **expression exists in patches of supporting cell progenitors in homozygous KI mice.** *In situ* hybridization of *Prox1* in E18.5 mice demonstrates the expression of *Prox1* in the spiral ganglia as well as in the supporting cells in wild-type (a,a’) and heterozygous KI mice (b, b’). In homozygous KI mice (c, c’), *Prox1* is expressed in patches of organ of Corti cells, except some continuity in the apex. *Prox1 in situ* signal also confirmed presence of spiral ganglia in the base of homozygous KI mice (a,b,c). Immunochemistry of Prox1 and tubulin shows patches of Prox1 positive organ of Corti cells in the base of cochlea which receive projection of the radial fibers to those patches in the homozygous KI mice (d, arrow in d). This supports formation of some supporting cells in clusters of organ of Corti cells. SC, supporting cells; ‘SC’, probable supporting cells; Spg, spiral ganglion cells. Bar indicates 100 µm.
(TIF)

Figure S6 **Loss of** ***Atoh1*** **enhances delamination of neurons with or without** ***Neurog1*** **expression.** At E13.5 *in situ* hybridization shows *Neurog1* expression only in the delaminating neurons in wild-type mice (a, arrow in a’) which is expressed both in delaminating neurons and in all vestibular epithelia in the homozygous KI mice (b, b') and in the delaminating neurons and in utricle and saccule in the *Atoh1* CKO mice (c, c’). *Bdnf* is expressed in hair cells in the vestibular end organs and apex of the cochlea with some expression in the delaminating neuroblasts in the wild-type cochlea (d,d’). *Bdnf* shows profound expression in delaminating neuroblasts near the utricle and saccule in homozygous KI mice (e,e’), comparable to *Atoh1* CKO mice (f, f’). The hair cells of the saccule are devoid of *Bdnf* expression in contrast to the *Neurog1* expression both in the homozygous KI (e,e’) and in *Atoh1* CKO mice (f,f’) which indicates *Bdnf* requires *Atoh1* for its upregulation in the saccular hair cells. Arrows indicate the delaminating neuroblasts and asterisks indicate the sensory epithelia. *Ntf3* remains nearly identical in wild-type (g, g’) homozygous KI mice (h, h’) and *Atoh1* CKO mice (i, i’). AC, anterior crista; Co, cochlea; HC, horizontal crista; PC, posterior crista; S, saccule; U, utricle. Bar indicates 100 µm.
(TIF)
