# Supplementary figures and images for: Expression of Neurog1 Instead of Atoh1 Can Partially Rescue Organ of Corti Cell Survival
Source: PLoS One. 2012 Jan 24;7(1):e30853. doi: 10.1371/journal.pone.0030853 (PMC3265522; doi:10.1371/journal.pone.0030853)

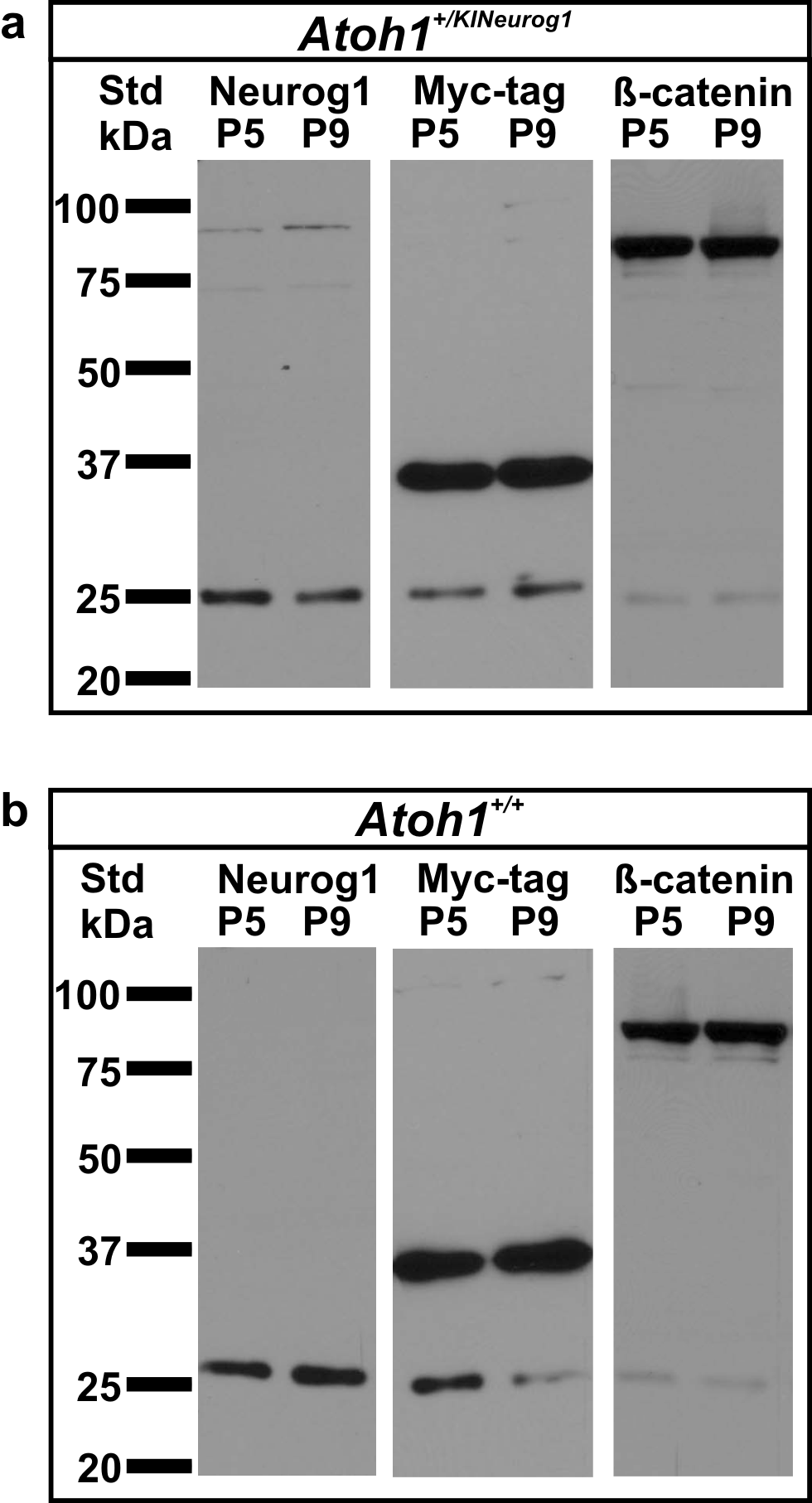

Supplement: Figure S2 — Neurog1 and Myc-tag Protein expression. Western blot analysis of Neurog1 and Myc-tag protein in the postnatal (P5 and P9) cerebella of heterozygous KI mice shows approximately correct molecular weight ∼25 and ∼36 kDa, respectively (a). However, wild-type littermates show similar sized bands, indicating some degree of non-specificity (b). β-Catenin antibody was used as the loading control in the western blot analysis (a,b). (TIF) [file pone.0030853.s002.tif]

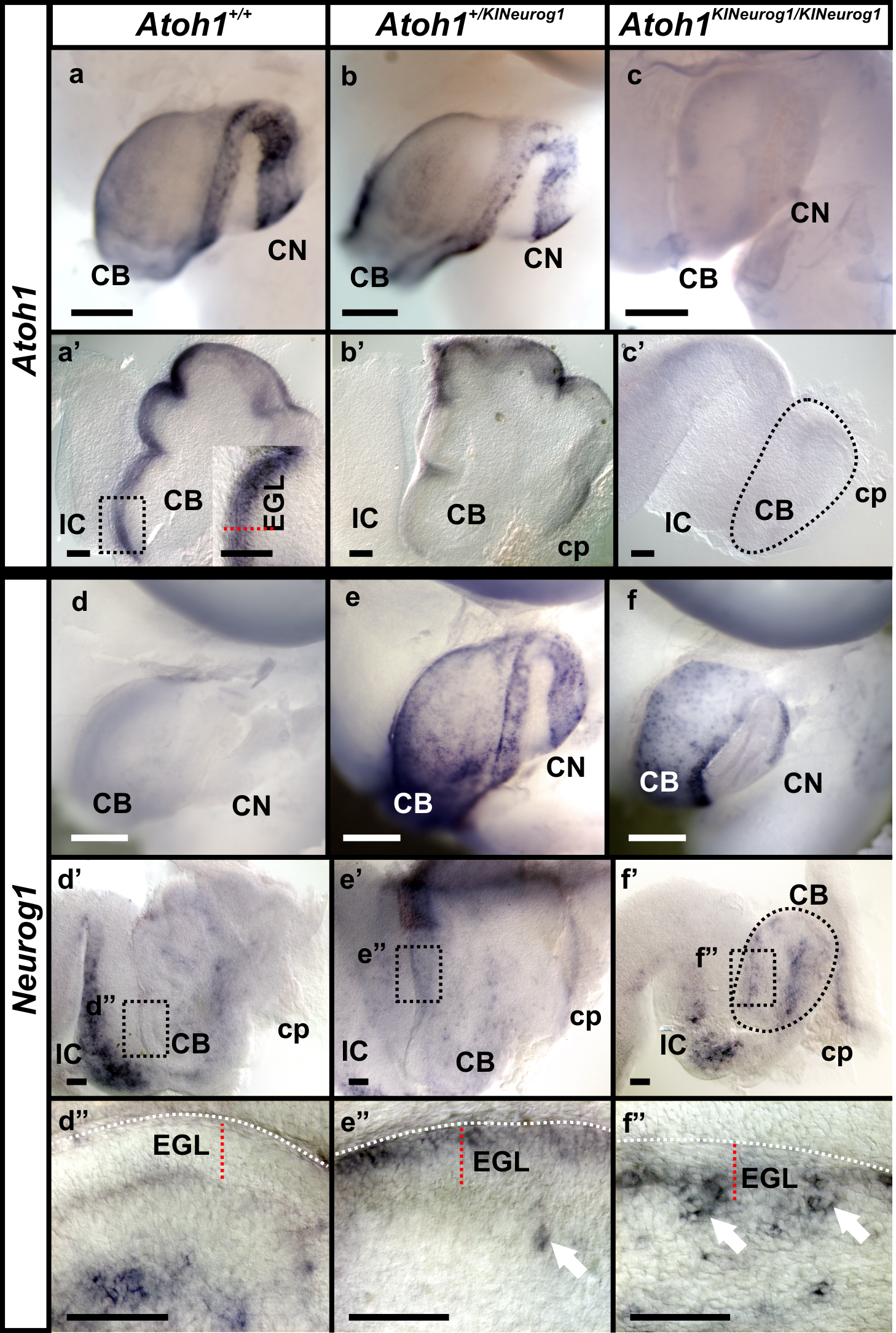

Supplement: Figure S3 — Atoh1 expression is replaced by Neurog1 in the cochlear nucleus and in cerebellum in Neurog1 KI mice. In situ hybridization shows downregulation of Atoh1 expression in the cochlear nucleus and in cerebellum of the heterozygous KI mice (b, b’) and complete absence in the homozygous KI mice (c, c’) compared to wild-type littermate (a, a’). Atoh1 is expressed in the proliferating precursors of the outer part of the external granule cell layer (insert in a’) which is maintained in some lobules in the heterozygous KI mice (b’). Neurog1 is completely absent in the cochlear nucleus and very faintly expressed in the deep nuclei of the cerebellum of wild-type mice without any expression in the cerebellar cortex (d-d”). In contrast, both heterozygous and homozygous KI mice show Neurog1 expression in the cochlear nucleus and in the proliferating external granule cell layer in cerebellum imitating the Atoh1 expression (e-e”, f-f”). Replacement of Atoh1 with Neurog1 successfully recapitulates Atoh1- pattern both peripherally (ear) and centrally (cochlear nucleus and cerebellum). The smaller sized cerebellum in homozygous KI mice is demarked with black dotted line in c’ and d’. Red dotted lines in d”, e”, f” demonstrate the area of external granule cell layer (EGL). CB, cerebellum; CN, cochlear nucleus; CP, choroid plexus; IC, inferior colliculus. Bar indicates 500 µm. (TIF) [file pone.0030853.s003.tif]

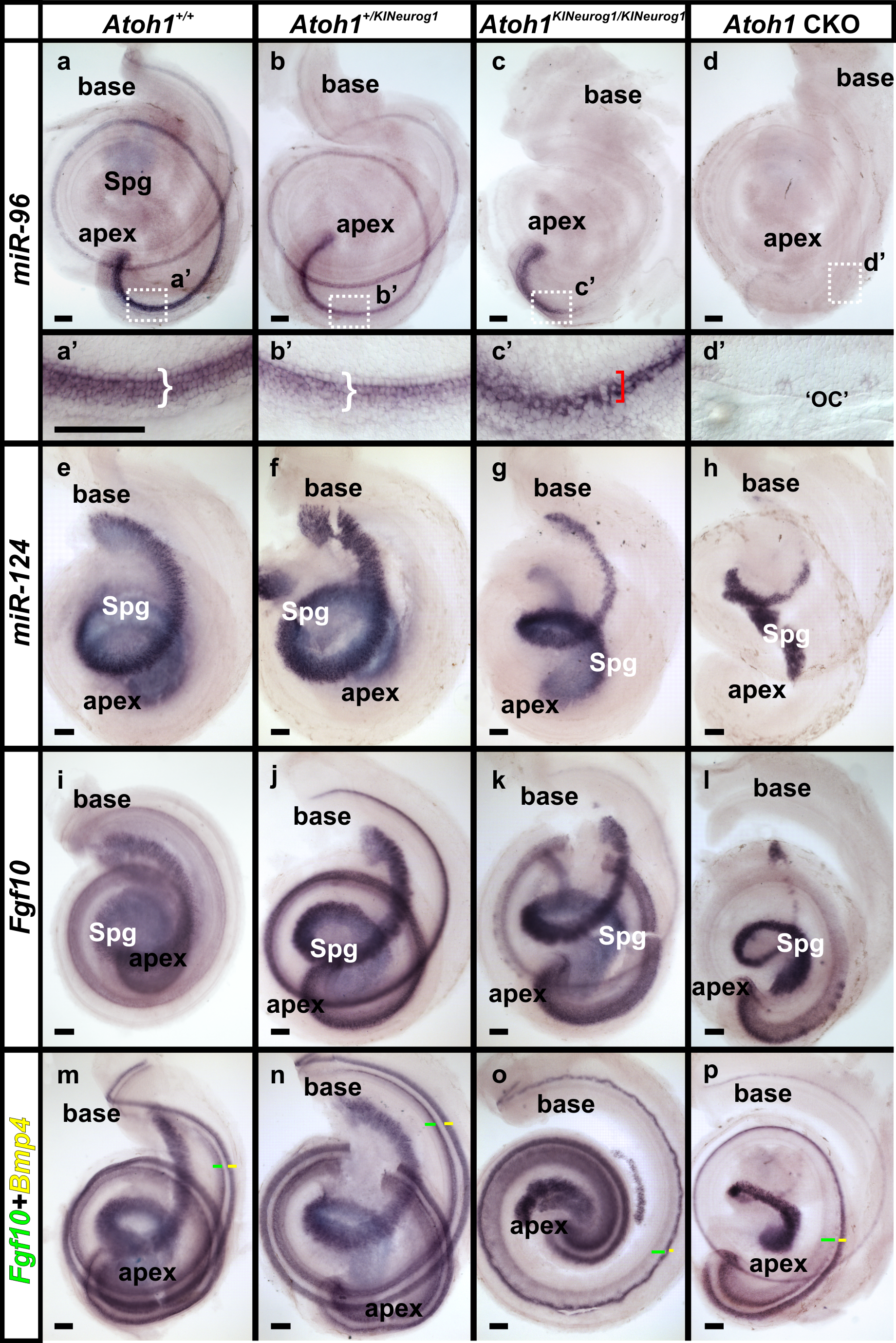

Supplement: Figure S4 — Neurog1 KI mice show basal turn spiral ganglia and enhanced organ of Corti gene expression compared to Atoh1 CKO mice. miR-96 in situ hybridization shows expression in the hair cells in both wild-type and heterozygous KI mice at E18.5 (a,a’,b, b’). miR-96 is expressed only in the apex of the homozygous KI cochlea whereas severely diminished in Atoh1 CKO mice (c,c’,d,d’). miR-124 is a neuronal marker expressed in all spiral ganglion cells (e-h). Homozygous KI mice retain neurons in the base that are almost absent in Atoh1 CKO mice (g,h). Neurog1 does not drive expression of miR-124 in hair cells. Another marker, Fgf10, is uniform in wild-type heterozygous knockin mice (i,j) and shows presence of neurons in the base of homozygous knockin cochlea (k) compared to Atoh1 CKO cochlea (l). Fgf10 is also expressed in the GER in wild-type and heterozygous KI mice (i,j). Homozygous KI mice show reduction of Fgf10 expression in the base, which is more profound in Atoh1 CKO (k,l). Another marker, Bmp4 is expressed in the Claudius cells defining the lateral (abneural) side of the developing organ of Corti. Simultaneous in situ hybridization of both Fgf10 and Bmp4 flank medial and lateral to the organ of Corti in wild-type and heterozygous KI cochleae (m,n). In homozygous KI mice, the patchy distribution of organ of Corti cells correlate with medial undulations of the Bmp4 expression in the base of the cochlea. Both Bmp4 and Fgf10 in situ signal are nearly absent in the Atoh1 CKO base (o,p). Note that the spiral ganglia in the homozygous KI mice are removed to allow complete non-overlapping mounting of the cochlea (o). Spg, spiral ganglia. ‘OC’, putative organ of Corti in Atoh1 CKO mice. ‘{‘ indicates the differentiated organ of Corti and ‘[‘ marks the presumed organ of corti in the homozygous KI mice. Green and yellow bar indicates Fgf10 and Bmp4 positive area, respectively. Bar indicates 100 µm. (TIF) [file pone.0030853.s004.tif]

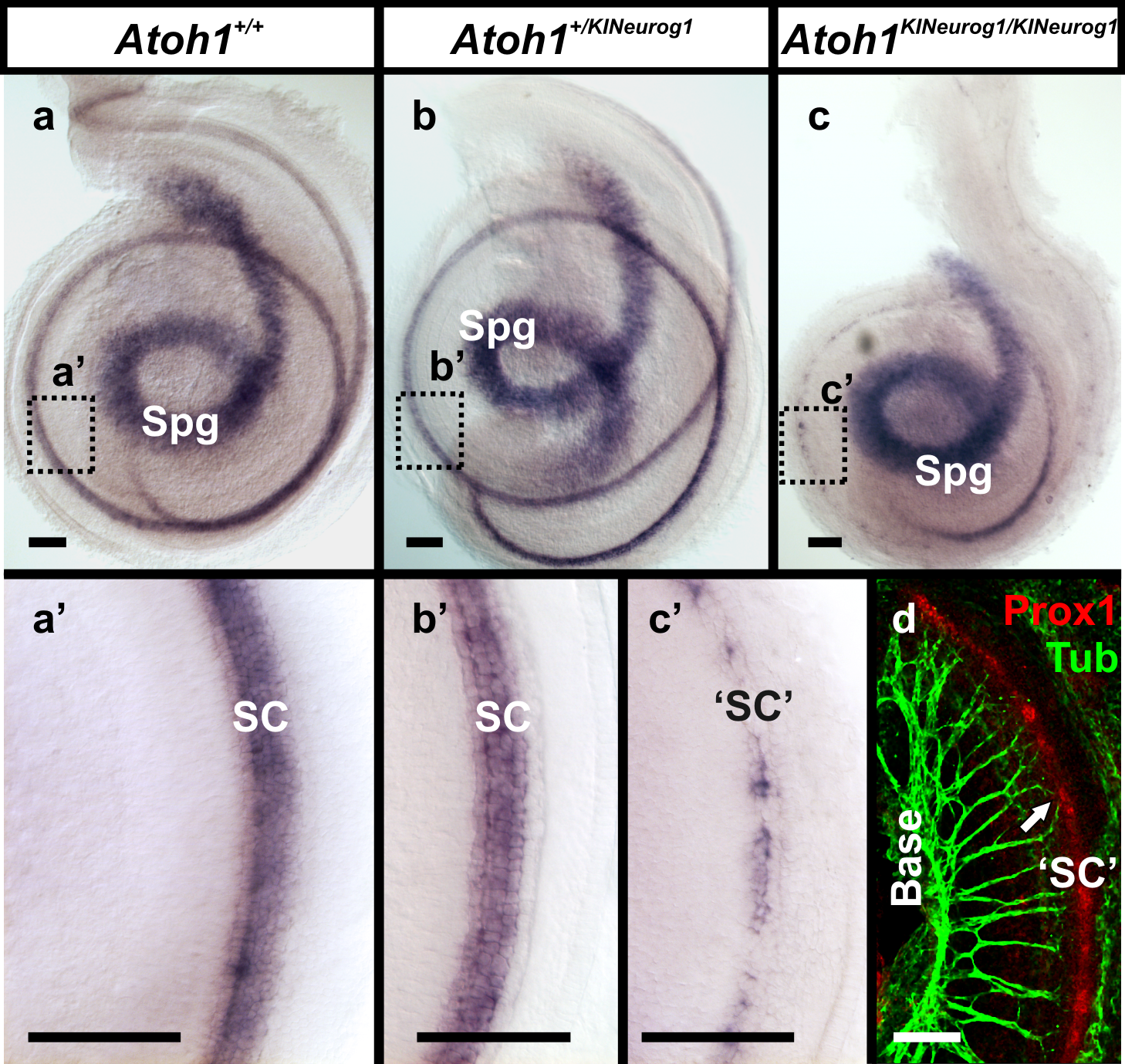

Supplement: Figure S5 — Prox1 expression exists in patches of supporting cell progenitors in homozygous KI mice. In situ hybridization of Prox1 in E18.5 mice demonstrates the expression of Prox1 in the spiral ganglia as well as in the supporting cells in wild-type (a,a’) and heterozygous KI mice (b, b’). In homozygous KI mice (c, c’), Prox1 is expressed in patches of organ of Corti cells, except some continuity in the apex. Prox1 in situ signal also confirmed presence of spiral ganglia in the base of homozygous KI mice (a,b,c). Immunochemistry of Prox1 and tubulin shows patches of Prox1 positive organ of Corti cells in the base of cochlea which receive projection of the radial fibers to those patches in the homozygous KI mice (d, arrow in d). This supports formation of some supporting cells in clusters of organ of Corti cells. SC, supporting cells; ‘SC’, probable supporting cells; Spg, spiral ganglion cells. Bar indicates 100 µm. (TIF) [file pone.0030853.s005.tif]

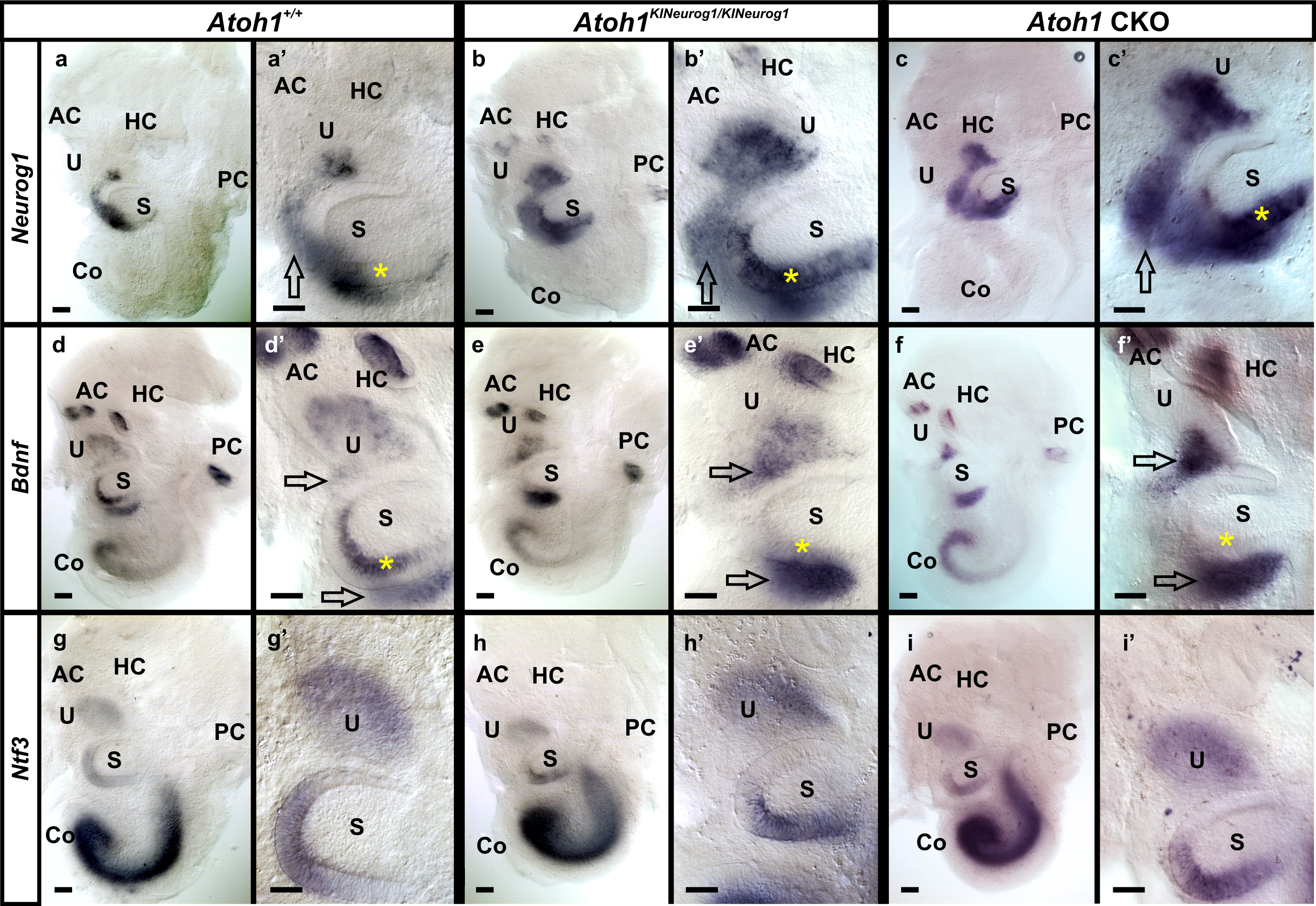

Supplement: Figure S6 — Loss of Atoh1 enhances delamination of neurons with or without Neurog1 expression. At E13.5 in situ hybridization shows Neurog1 expression only in the delaminating neurons in wild-type mice (a, arrow in a’) which is expressed both in delaminating neurons and in all vestibular epithelia in the homozygous KI mice (b, b') and in the delaminating neurons and in utricle and saccule in the Atoh1 CKO mice (c, c’). Bdnf is expressed in hair cells in the vestibular end organs and apex of the cochlea with some expression in the delaminating neuroblasts in the wild-type cochlea (d,d’). Bdnf shows profound expression in delaminating neuroblasts near the utricle and saccule in homozygous KI mice (e,e’), comparable to Atoh1 CKO mice (f, f’). The hair cells of the saccule are devoid of Bdnf expression in contrast to the Neurog1 expression both in the homozygous KI (e,e’) and in Atoh1 CKO mice (f,f’) which indicates Bdnf requires Atoh1 for its upregulation in the saccular hair cells. Arrows indicate the delaminating neuroblasts and asterisks indicate the sensory epithelia. Ntf3 remains nearly identical in wild-type (g, g’) homozygous KI mice (h, h’) and Atoh1 CKO mice (i, i’). AC, anterior crista; Co, cochlea; HC, horizontal crista; PC, posterior crista; S, saccule; U, utricle. Bar indicates 100 µm. (TIF) [file pone.0030853.s006.tif]
